# Construction of the Node—place—Jobs-housing model: Analysis of employment-residential ratio in subway station areas of Shenzhen, China’s highest construction density zone

**DOI:** 10.1371/journal.pone.0337576

**Published:** 2025-12-05

**Authors:** Fang Liu, Jingyi Zhang, Hao Geng, Yusong Zhu, Yanmei Zhu, Jiahao Zhou

**Affiliations:** 1 State Key Laboratory of Subtropical Building and Urban Science, School of Architecture and Urban Planning, Benyuan Design Research Center, Shenzhen University, Shenzhen, China; 2 Faculty of Humanities and Arts, Macau University of Science and Technology, Macau, China; 3 School of Architecture, Southeast University, Nanjing, Jiangsu, China; Chang'an University, CHINA

## Abstract

Transit-oriented development (TOD) is widely recognized as a land development mode designed to integrate residential and employment spaces, fostering a balanced distribution of jobs and housing while reducing reliance on motorized transportation. Nonetheless, some scholars argue that small-scale TOD station areas should relax their self-sufficiency and jobs-housing balance requirements, creating a conceptual contradiction. To date, no clear consensus exists on how the employment-residential ratio relates to factors such as transportation supply, land use patterns, and the degree of TOD development, often referred to as TODness. Addressing this gap, this study examines subway station areas within the highest-density construction zone of Shenzhen, China, and extends the well-established node-place (NP) framework into a three-dimensional NP-jobs-housing (NPJ) model. The employment-residential ratio exhibits a positive linear relationship with public transportation supply, land development intensity, and development density. Conversely, it shows a negative linear relationship with the diversity of land construction and development. The TODness of station areas has a U-shaped effect on the employment-residential ratio, with a threshold value of 0.775 marking the inflection point. Incorporating the employment-residential ratio into the analysis enables classification of TOD station areas into four types, with significant jobs-housing imbalances observed only in areas with either low (Place value mean ≤ 0.146) or high (Place value mean ≥ 0.771) land development intensity. Prioritizing residential land development, followed by commercial and office space construction in later stages, better aligns with TOD principles. In the later stages of development, the employment-residential ratio tends toward employment dominance; however, increasing diversity of development emerges as an effective strategy to counteract imbalance. Overall, this research advances understanding of jobs-housing distribution within the TOD framework and provides insights for guiding land use planning and development adjustments based on station typology and TOD maturity.

## 1 Introduction

The balance between jobs and housing represents a core objective of urban planning, encompassing coordinated efforts in both land use and transportation supply [1], with the ultimate goal of aligning land development and transit systems to prevent the waste of urban construction resources and to promote sustainable urban growth [2]. This principle is embedded in the widely recognized “smart growth” planning theory [3], particularly within the framework of the public transit-oriented development (TOD) mode [4,5], which is considered an effective approach for achieving a reasonable employment-residential ratio in high-density urban areas [6–9]. Despite its importance, empirical research examining the employment-residential ratio within TOD station areas remains limited [10], largely due to two factors. First, in the TOD planning framework, the station catchment area, defined as land within a 500-meter radius (approximately a 10-minute walk) from a TOD station, covers about 0.785 square kilometers [11], which is significantly smaller than a transportation analysis zone, census tract, or street unit, within which the jobs-housing balance is typically assessed [12]. For example, in Hangzhou, the average transportation analysis zone covers 2.35 square kilometers [13], while in China’s administrative planning, the jobs-housing balance is often evaluated at the street level; in Beijing, the average street, as the basic administrative unit, spans approximately 9.95 square kilometers [14]. Second, research indicates that in high-density, mixed-use TOD station areas, overall commuting costs decrease, making deviations from the theoretical employment-residential ratio both normal and acceptable [15,16]. Consequently, determining an appropriate employment-residential ratio that is compatible with land use patterns and transportation supply is essential [11]; however, existing typological studies still lack clarity regarding the ratios for different types of TOD station areas [10], thereby limiting the precision and effectiveness of detailed land-use regulatory planning.

If the goal is to investigate the employment-residential ratio in TOD station areas in a manner that harmonizes land use with transportation supply, then measurement methods and indicators derived from micro-level pedestrian commuting data, such as excessive commuting, theoretical maximum commuting distance [17], random average commuting distance [18], proportional matching commuting [19], normalized location-weighted landscape index [16], and local co-location quotient (LCLQ) [20], are often unsuitable, as regulatory detailed planning for station areas requires a mesoscopic statistical perspective to capture the pairing relationships among land use, transportation supply, and the employment-residential ratio. Applying micro-level pedestrian commuting methods at this scale would necessitate complex data transformations. In contrast, the classic “node-place model” (NP Model) provides a well-established mesoscopic-level analytical framework, in which transportation supply (Node) is treated as a node attribute representing the public transit capacity of the station area, including subways, buses, and private vehicle access, while land use (Place) is defined as a place attribute capturing road morphology, development intensity, and the degree of mixed-use development [5,21–23]. By extending the NP Model to include indicators representing the employment-residential ratio as a third dimension (Jobs-Housing), it becomes possible to explore the compatibility relationships among land use, transportation supply, and the employment-residential ratio. However, it must be acknowledged that the NP Model is inherently a static framework, originally designed to examine the correspondence between Node and Place attributes, to classify TOD station areas based on these relationships [24,25], and to evaluate the degree of TOD development, or TODness [5,26]. Consequently, even with the integration of the jobs-housing dimension, the resulting NP-jobs (NPJ) housing model remains a static analytical tool and, as such, lacks the capacity to establish or explain causal relationships among its three dimensions.

Accordingly, this study seeks to develop the NPJ model as a framework for examining the compatibility relationships among Node values, Place values, and Jobs-Housing values across different types of TOD station areas, while also investigating the interaction effect of TOD development degree (TODness) on Jobs-Housing dynamics within this framework. The research aims to establish a set of reference control values for land use, transportation supply, and employment-residential ratios applicable to TOD station areas, to identify appropriate employment-residential ratios for various TOD typologies, and to analyze the patterns and driving factors underlying changes in these ratios as TODness increases.

The research process begins by selecting indicators from the three dimensions of NPJ-Housing, guided by the requirements of land regulatory detailed planning and existing literature. Correlation analysis is then applied to validate and refine these indicators, forming the basis for constructing the NPJ model. Subsequently, Node values, Place values, Jobs-Housing values, and TODness are calculated for the station area samples, followed by an examination of the key factors influencing Jobs-Housing values. In the second stage, cluster analysis is employed to classify station areas according to the NPJ model, with the numerical characteristics of each category summarized. A comparative assessment is then conducted between TOD station area types identified under the NP model and those derived from the NPJ model to evaluate their correspondence. The third stage examines various station area samples within the study’s urban scope, analyzing the land use characteristics and spatial distribution patterns of each type to support urban planners in rapidly identifying appropriate construction typologies and Jobs-Housing values (employment-residential ratios) for TOD station areas. Finally, the fourth stage applies polynomial regression to model the influence of TODness on variations in Jobs-Housing values, thereby providing a decision-making reference for determining suitable employment-residential ratios across different stages of TOD development.

## 2 Literature review

### 2.1 Measurement methods for jobs-housing balance

The jobs-housing balance is defined as “the degree of equilibrium between the distribution of employment opportunities and the distribution of workers within a given area” [27], with its core objective being to align employment opportunities and housing supply to reduce commuting times and alleviate traffic congestion [1], thereby reflecting a region’s capacity for self-sufficiency. The theoretical foundation of this concept lies in the Independence Index proposed by Thomas in 1969, which quantifies the proportion of residents who both live and work locally, serving as a measure of self-sufficiency [28]. Cervero [29] argues that the uniformity of employment and population distribution, expressed through the ratio of jobs to housing, indicates only the potential for achieving balance, while actual self-sufficiency involves two dimensions: the matching of employment and housing (employment-residential ratio) and the spatial proximity of jobs to residences [14].

A widely used metric for assessing the employment-residential ratio is the balance index method developed by Peter Gordon and colleagues in the 1980s [30], which calculates the ratio of jobs to housing units within a given area [31]. Values within the range of 0.8 to 1.2 are generally regarded as indicative of balance [8], and the method is frequently employed in urban planning to determine land development proportions. However, its application is subject to the modifiable areal unit problem (MAUP) [32], as results may vary with changes in the size of the analyzed region, and it represents an idealized figure that should be adjusted according to the city’s average jobs-housing ratio to ensure practical relevance [33]. Similarly, the jobs-to-workers ratio (JWR) and its variant, the Adjusted JWR (AJWR), calculated by dividing the number of jobs by the number of employed residents in an area [34], also encounter MAUP-related limitations in both research and practice [35]. Core indicators in such models typically include the number of housing units, the number of job locations, and the proportion of housing located near workplaces [36]. In contrast, the LCLQ method places greater emphasis on measuring spatial proximity between jobs and housing, though its effectiveness is contingent upon a relatively balanced employment-residential ratio, as proximity analysis becomes meaningful only when the number of jobs and housing units is approximately equivalent.

Balance measures that emphasize the spatial proximity of employment to housing also encompass methods such as excessive commuting, theoretical maximum commuting distance [17], random average commuting distance [18], and proportional matching commuting [19], which examine the distance between residential and workplace locations from the standpoint of individual travel behavior [36] to determine whether a region has attained a self-sufficient state. The key indicators in these models typically include the number of urban areas, the number of destinations, the resident population, the number of local employment opportunities, inter-regional travel costs, and the frequency of cross-regional commuting [37]. While these approaches are valuable for assessing the rationality of urban structures, industrial distribution, and settlement patterns from a city-wide perspective, thereby supporting large-scale transportation route planning and network design, they offer limited utility for making finer-grained decisions concerning land development intensity, land use diversity, or the appropriate scale of transportation infrastructure at the station-area or neighborhood level.

### 2.2 The role of the NP model and the feasibility of expanding the NPJ model

TOD is a land development strategy that leverages public transportation to guide the planning of areas surrounding transit stations, fostering multifunctional, mixed-use, compact, and high-density communities [38,39]. Achieving a rational employment-residential ratio is both a central objective of the TOD mode and a desired outcome of the land use mix that this strategy promotes [40]. However, conventional measures such as the balance index, jobs-to-workers ratio, and excessive commuting are ill-suited for evaluating land use and planning configurations at the fine-grained urban scale characteristic of TOD station areas. Moreover, because employment-residential ratios in such areas often deviate from an ideal balance, the LCLQ method is likewise unsuitable for studies seeking to determine a reasonable employment-residential ratio specifically within TOD station area contexts.

**Fig 1:**
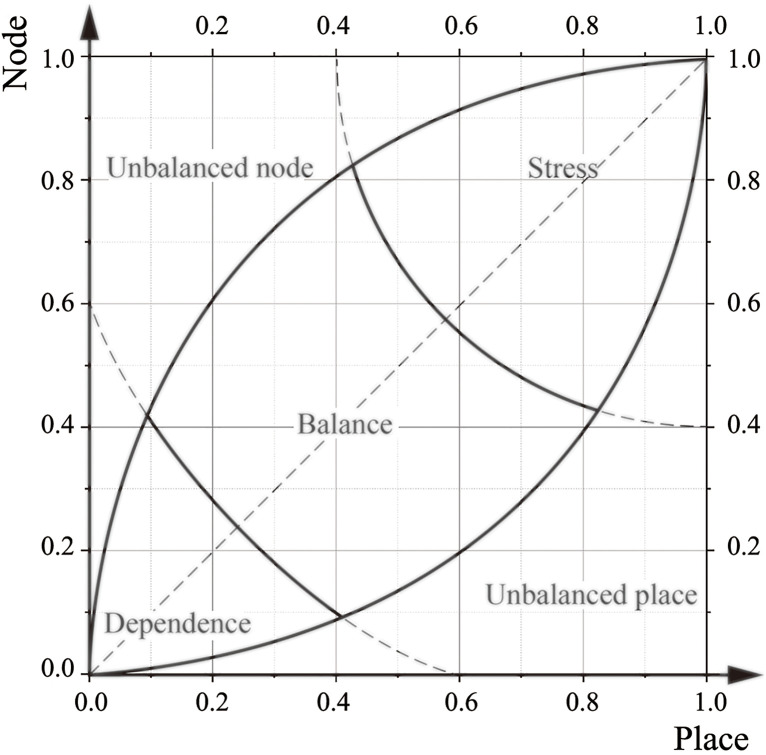
Schematic diagram of the Node-Place model (The arc positioning principle in the model is detailed in S1 File).

At the beginning of the 21st century, Bertolini introduced the NP model, a typological framework specifically designed for TOD analysis [25,41] (Fig 1). In this model, the Y-axis (Node axis) represents the external transportation capacity of the station area [5,12,42], while the X-axis (Place axis) reflects the land use capacity within the station area to accommodate diverse activities [5,12,42]. The NP model serves as a visual and analytical tool for evaluating the degree of alignment, or matching effectiveness, between land use and transportation supply in TOD station areas. Based on this matching relationship, the model categorizes station areas into five types: Balance, Dependency, Stress, Unsustained Node, and Unsustained Place [24,25,43]. However, the model’s assessment of land-transportation construction effectiveness is inherently statistical and typological, representing a snapshot of development at a specific point in time. The classifications it generates are therefore applicable only to station area samples drawn from the same construction context and temporal cross-section, meaning that all analyzed stations must share comparable developmental and temporal conditions for meaningful typological comparison.

Research on the NP model has largely concentrated on two main directions. The first involves maintaining the model’s original two-dimensional structure while developing calculation methods for TODness, grounded in the NP model’s statistical framework, to enable comparative evaluation of TOD development degree across station areas [44]. The second aligns more closely with the objectives of the present study and entails expanding the NP model into a three-dimensional framework, referred to as the NPX model [22], in which the “X” dimension is defined according to specific research objectives. This approach has yielded variations such as the NPV (Vitality, Vibrancy) Model [5,42], the NPD (Design) Model [5,45,46], and the NPF (Functionality) Model [44]. Building on the NPX framework, researchers have applied cluster analysis to produce revised TOD classifications that incorporate the effects of the added dimension, while also categorizing and summarizing the matching relationships among land use, transportation supply, and the specific characteristics represented by “X” [21,26,47,48].

In the application and evolution of the NP model, relatively little attention has been given to examining it from the perspective of the jobs-housing balance. Yet, the relative appropriateness of the employment-residential ratio is not only an inherent objective of the TOD land development mode but also a critical parameter to be defined in the regulatory detailed planning of urban areas [5,40]. Drawing on the methodological framework and extension principles of the NPX model developed in recent years, it is both theoretically sound and methodologically feasible to advance the NP model into a NPJ model.

### 2.3 The logical relationship between employment-residential ratio and Node, Place, and TODness in the TOD framework

The jobs-housing relationship pursued in TOD station areas is not one of absolute self-sufficiency or perfect balance, but rather the attainment of a reasonable employment-residential ratio that is compatible with prevailing land use and transportation supply conditions [11]. Furthermore, studies on TOD development modes and applications of the NP model have identified a degree of drift in station-area transportation supply [47], wherein the resident workforce may commute from other stations, and the commuters served by the station may not necessarily work within the station area itself. Nevertheless, such dynamics do not preclude the examination of compatibility between land use and transportation supply, nor do they hinder the assessment of how the employment-residential ratio aligns with these two elements. A fundamental prerequisite for applying the NP model is that all samples must be drawn from station areas sharing the same construction context and temporal cross-section. Within such a stable regional framework, the research seeks to determine the compatibility relationships among the three components, thereby providing valuable insights to inform planning and regulatory decision-making for land use allocation and adjustment.

Establishing the significance of constructing the NPJ model first requires determining whether a logical relationship exists between the employment-residential ratio and the Node value, Place value, and TODness. As a land development approach, the TOD mode aims to maximize the use of public transportation to improve land use efficiency, thereby promoting the growth of surrounding commercial and residential areas [49]. This approach has been validated as effective in achieving a reasonable employment-residential ratio in densely populated urban contexts [6–9]. Both work and residential behaviors depend on the spatial framework shaped by regional land development and the availability of transportation supply. Consequently, it can be qualitatively inferred that the employment-residential ratio is correlated with the Node value and Place value. TODness, which reflects the degree of TOD development, is derived from these two values and indicates the extent of TOD implementation in station areas as well as their potential for sustainable growth [5,12]. Its calculation incorporates the Node value, Place value, and the inverse variance between the two (formula provided in Section 4.5), thereby representing the synergistic development of land and transportation. In practice, shifts in residential and employment demand can exert a reverse regulatory influence on transportation supply and land development. However, such discussions on employment-residential behaviors and ratios typically emerge only after a station area reaches a certain stage of development, when it is capable of supporting both living and working spaces alongside adequate transportation capacity. Empirical research has shown that the development of station areas, such as those served by light rail, can reshape the spatial distribution of jobs and housing, thereby altering the employment-residential ratio [50]. Furthermore, studies controlling for confounding factors such as urban structure [51] and regional socioeconomic conditions [52] have found that land development and built environment characteristics significantly affect the jobs-housing ratio [16,53]. Taken together, these findings qualitatively suggest that TODness influences the employment-residential ratio in station areas.

## 3 Study area, sample selection, and data collection

### 3.1 Study area and sample selection

This study focuses on Shenzhen’s highest-density construction area, Density Zone 1, as the urban region for analysis. Characterized by rapid urbanization, substantial population growth, and a complex transportation network, Density Zone 1 is considered representative of high-density urban districts worldwide [54,55]. Within this zone, the TOD land development model has been fully implemented, with transportation infrastructure and land development reaching an advanced stage of completion. By the selected temporal cross-section (December 2024, the data collection period), both land development and subway operations in the sampled station areas had stabilized. Previous research indicates that the outcomes of intensive and coordinated development in this region significantly surpass those achieved in comparable large cities and regions [5,54]. Furthermore, the Shenzhen Urban Planning Standards and Guidelines (2021) [56] provide zoning regulations for construction land density that define the development context and baseline intensity for Density Zone 1. These guidelines ensure that all subway station areas within the zone share consistent socioeconomic conditions, development benchmarks, and construction backgrounds.

Shenzhen is a typical polycentric city, a structural form consistent with the development frameworks adopted by many other high-density cities, including San Francisco in the United States [57]; Paris, Lyon, and Marseille in France [58]; Randstad in the Netherlands [59]; Sydney in Australia [60]; and major urban centers in Turkey and China since the early 21st century [61,62]. Density Zone 1 geographically encompasses the central urban areas of several planned districts [63] and is characterized by a high concentration of urban handicrafts, commerce, office spaces, and residential areas, while excluding heavy manufacturing [55] This profile aligns with the core-area characteristics of other polycentric metropolitan regions and produces commuting patterns similar to those observed in comparable cities [5,64] Notably, Density Zone 1 contains 72 completed subway station areas, representing all five categories in the NP model. This distribution enables a direct comparison between older and newer station area classifications following the development of the NPJ model (Fig 9a). Collectively, these features make Shenzhen’s highest-density construction area an exemplary case for developing the NPJ model and for examining the interrelationships between residential-employment ratios, traffic supply, land use, and TODness.

**Fig 2:**
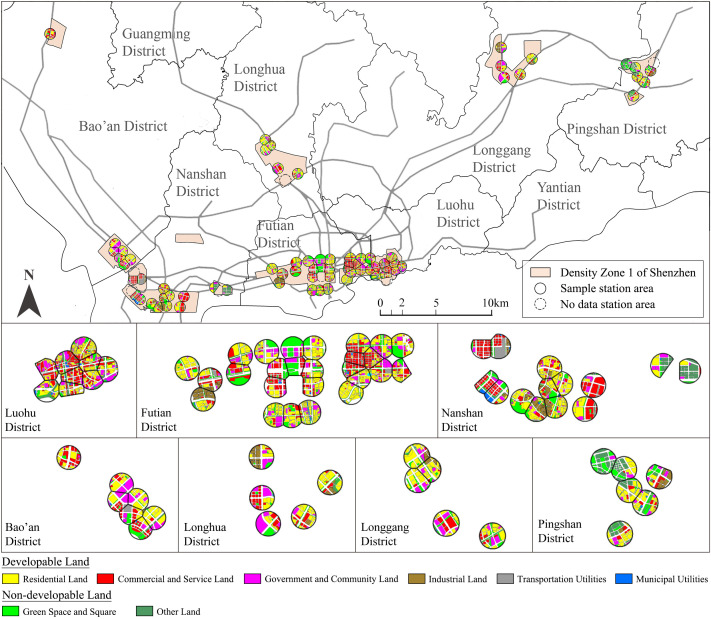
Distribution of all samples. Base map data from OpenStreetMap. The figure is a simplified representation for illustrative purposes.

According to the Shenzhen Construction Land Density Zoning Guideline Map published by the Planning and Natural Resources Bureau of Shenzhen Municipality in 2021, Density Zone 1 covers the urban core areas of seven natural planning districts in Shenzhen (Fig 2) [56]. As of December 2024, this zone contains 79 operational subway stations; however, four stations were excluded due to incomplete construction, and three intercity high-speed railway stations were omitted because their population data is influenced by intercity travel and cannot be reliably captured. Consequently, the final research sample comprises 72 subway station areas (Fig 2).

In this study, a TOD station area is defined as a zone centered on a subway, light rail, or bus rapid transit station, with a radius of 400–800 m (a 5–10 minute walk) [40,65]. Specifically, each subway station area was delineated as a 500 m Euclidean radial zone [11]. While official Chinese guidelines recommend a radius of 500–800 m for subway station areas [66], several scholars contend that the effective catchment radius for most metro stations is less than 800 m [67], and numerous studies have also adopted a 500 m standard [68]. This distance is consistent with land-use intensity regulations issued by the Shenzhen Planning and Natural Resources Bureau for areas surrounding subway stations, ensuring uniformity in the construction context of the selected samples [57]. When adjacent subway station areas overlapped, defined as having a straight-line distance of less than 1 km between stations, the Voronoi polygon method was employed to delineate boundaries and prevent spatial duplication [69,70].

### 3.2 Sources of data collection

This study involves the systematic collection of data across three key dimensions: transportation supply (Node), land use (Place), and employment-residential ratio (Jobs-housing), with the specific indicators for each dimension outlined in Table 1. The Node dimension represents the traffic supply capacity of subway station areas, encompassing subway service capacity, bus service capacity, and private vehicle carrying capacity. The Place dimension reflects the station area’s ability to accommodate diverse urban activities, incorporating factors such as land development intensity, urban construction patterns, land development density, and the diversity of construction and development. The Jobs-housing dimension evaluates the employment-residential ratio of the station areas, measured through two indicators: the employment-to-residential area ratio and the employment-to-residential population ratio.

**Table 1:** Indicators of Node–Place–Jobs-Housing in this work.

Dimension	Category	Code	Indicators	Description and data collection	Data source
Node	Subway supply capacity	S1	Betweenness centrality	${\text{C}}_{\text{betweenness}}(\text{i})=\sum_{\text{kt}}\;\frac{\sigma_{\text{kt}}(\text{i})}{\sigma_{\text{kt}}},$ where $\sigma_{\text{kt}}(\text{i})$ is the number of shortest paths through i between any two subway stations k and t, and $\sigma_{\text{kt}}$ is the total number of paths between k and t. The betweenness centrality of each station was calculated based on the overall metro network map of Shenzhen provided on the official website of Shenzhen Metro Company (Operations Department).	https://www.szmc.net/index/
		S2	Number of directions	For each station, the initial value of direction is 2, and for each additional line that can be transferred, the direction increases by 2. The data comes from the official website of Shenzhen Metro Company (Operations Department).	https://www.szmc.net/index/
		S3	Departure interval	The average train service frequency at the station. The data comes from the official website of Shenzhen Metro Company (Operations Department).	https://www.szmc.net/index/
		S4	Operating time	How long does subway station operates daily? The data comes from the official website of Shenzhen Metro Company (Operations Department).	https://www.szmc.net/index/
		S5	Number of compartments	The number of compartments passing through the metro station per rush hour. The data comes from the official website of Shenzhen Metro Company (Operations Department).	https://www.szmc.net/index/
	Bus supply capacity	B1	Density of bus stop	B1=N/S, where N is the number of bus stops within the subway station area, and S is the total area of the station area. The data on the number of bus stops comes from the Baidu Maps Open Platform.	https://lbsyun.baidu.com/apiconsole/center#/home
		B2	Bus operating time	How long does the bus stop operates daily? The daily operating hours of the bus stops are obtained from the official website of the Shenzhen Municipal Transportation Bureau based on the respective routes.	http://jtys.sz.gov.cn/
	Private car carrying capacity	T1	Density of the parking lot	T1=C/S, where C is the number of parking lots within the subway station area, and S is the total area of the station area. The data on the number of car lots comes from the Baidu Maps Open Platform.	https://lbsyun.baidu.com/apiconsole/center#/home
Place	Land development scale	G1	Floor area ratio	$G\text{1}=\sum_{i}^{n}B_{i}/(M-W)$, where M is the total area of the station area, and W is the water area of the station area, $B_{i}$ is the area of the building i in the station area, n is the number of buildings in the station area. $B_{i}$ and W data are sourced from the public open version of the ‘Detailed Planning Map’ on the Data Open Platform of Shenzhen Municipal Planning and Natural Resources Bureau.	http://pnr.sz.gov.cn/d-xgmap/
		G2	Population size	G2=P/S, where P is the population number within the subway station area, and S is the total area of the station area. The data on population numbers comes from the Baidu Heat Map.	https://map.baidu.com
	Urban construction form	U1	Total road length	Total road length of the subway station area. The data comes from the Baidu Maps Open Platform.	https://lbsyun.baidu.com/apiconsole/center#/home
		U2	Number of intersections	Intersection number of the subway station area. The data comes from the Baidu Maps Open Platform.	https://lbsyun.baidu.com/apiconsole/center#/home
	Land development density	D1	Building density	$D\text{1}=\sum_{i}^{n}S_{i}/(M-W)$, where M is the total area of the station area, and W is the water area of the station area, $S_{i}$ is the first floor area of the building i in the station area, n is the number of buildings in the station area. $S_{i}$ and W data are sourced from the public open version of the ‘Detailed Planning Map’ on the Data Open Platform of Shenzhen Municipal Planning and Natural Resources Bureau.	http://pnr.sz.gov.cn/d-xgmap/
		D2	POI density (point of interest density)	D2=I/S, where I is the number of POIs within the subway station area, and S is the total area of the station area. I data is sourced from the public open version of the ‘Detailed Planning Map’ on the Data Open Platform of the Shenzhen Municipal Planning and Natural Resources Bureau.	https://map.baidu.com
	Construction and development diversity	M1	Land use mixture	Land use mix degree measures the equilibrium of land use proportions through the standardized ratio of the range and the mean. The principle is that as the proportions of different land uses become closer to each other, the land use mix degree approaches 1, thereby suppressing the emergence of extreme dominant functions. $M\text{1}=\text{1}-\frac{\left(a-\frac{b}{d}\right)-\left(a-\frac{c}{d}\right)}{\text{2}}$, where $a=MAX(D_{\text{1}},D_{\text{2}},D_{\text{3}},D_{\text{4}},D_{\text{5}},D_{\text{6}});b=MIN(D_{\text{1}},D_{\text{2}},D_{\text{3}},D_{\text{4}},D_{\text{5}},D_{\text{6}})$; $c=(D_{\text{1}}+D_{\text{2}}+D_{\text{3}}+D_{\text{4}}+D_{\text{5}}+D_{\text{6}})/6$; $d=P_{\text{1}}+P_{\text{2}}+P_{\text{3}}+P_{\text{4}}+P_{\text{5}}+P_{\text{6}}$, D1 to D6 represent the residential land, commercial and service land, government and community land, industrial land, and transportation utilities, municipal utilities, respectively. The urban land types D1-D6 are sourced from “SHENZHEN URBAN PLANNING STANDARDS AND GUIDELINES (2021) [56].The data from D1 to D6 is sourced from the public open version of the ‘Detailed Planning Map’ on the Data Open Platform of Shenzhen Municipal Planning and Natural Resources Bureau.	http://pnr.sz.gov.cn/d-xgmap/
		M2	Land use entropy	Land use mix entropy quantifies the diversity and uniformity of land use by calculating the weighted sum of the negative logarithm of the proportions of various land uses. The underlying principle is that as the number of land use types increases and their proportions become more similar, the entropy value increases, indicating a higher degree of functional interweaving. $M\text{2}=-\sum_{i}^{n}\;P_{i}log(D_{i})$, used to measure the diversity of land use, where $D_{i}$ is the proportion of the land use type i, n is the number of land use types. $D_{i}$ can be proportions of residential land, commercial and service land, government and community land, industrial land, and transportation utilities, municipal utilities. The data of Di is sourced from the public open version of the ‘Detailed Planning Map’ on the Data Open Platform of Shenzhen Municipal Planning and Natural Resources Bureau.	http://pnr.sz.gov.cn/d-xgmap/
		M3	Building mixed entropy	Building mix entropy quantifies the diversity and uniformity of building functions by calculating the weighted sum of the negative logarithm of the proportions of various building types. The underlying principle is that as the number of building types increases and the proportions of different functional areas become more similar, the entropy value rises, indicating a greater degree of integration among building functions.$M\text{3}=-\sum_{i}^{n}\;P_{i}log(D_{i})$, where $D_{i}$ is the proportion of the building type i, n is the number of building types. $D_{i}$ can be residential building, commercial and service building, government and community building, industrial building, transportation buildings and municipal buildings. The data of Di is sourced from the public open version of the ‘Detailed Planning Map’ on the Data Open Platform of Shenzhen Municipal Planning and Natural Resources Bureau.	http://pnr.sz.gov.cn/d-xgmap/
Jobs-Housing	Employment -residential ratio	JH1	employment – residential area ratio	Ratio of employment building area to residential building area in subway station area. The data for employment building area and residential building area is sourced from the public open version of the ‘Detailed Planning Map’ on the Data Open Platform of Shenzhen Municipal Planning and Natural Resources Bureau.	http://pnr.sz.gov.cn/d-xgmap/
		JH2	employment – residential population ratio	Ratio of population working to residents in subway station area. The working and residential population data were collected based on the station area range from the big data platform ‘Digital Observation’. A total of 7 days (Monday to Sunday) were collected, with cross-sectional data gathered at 6 time points each day (7:00, 9:00, 12:00, 15:00, 19:00, and 22:00), and then averaged.	https://www.swguancha.com/

Transportation supply data were obtained from the official websites of the Shenzhen Metro Company (Operations Department) (https://www.szmc.net/), the Shenzhen Municipal Transportation Bureau (http://jtys.sz.gov.cn/), and the Baidu Maps Open Platform (https://lbsyun.baidu.com/apiconsole/center#/home). Land use data, including floor area ratio, building density, point of interest (POI) density, land use mix, land use entropy, and building mix entropy, were sourced from the “Detailed Planning Map” on the Data Open Platform of the Shenzhen Municipal Planning and Natural Resources Bureau (http://pnr.sz.gov.cn/d-xgmap/). Population size data were derived from the Baidu Heat Map (https://map.baidu.com), while data on total road length and number of intersections were acquired from the Baidu Maps Open Platform (https://lbsyun.baidu.com/apiconsole/center#/home). Employment-to-residential area ratio data were obtained from the “Detailed Planning Map” on the same Data Open Platform, and employment-to-residential population ratio data were sourced from the big data platform “Digital Observation” (https://www.swguancha.com/).

Furthermore, the cartographic base maps for the study area (Shenzhen) used in Figs 2, 10, and A3 were derived from open data provided by OpenStreetMap contributors (https://www.openstreetmap.org), available under the Open Database License (ODbL). These maps were adapted by the authors for illustrative purposes.

**Fig 3:**
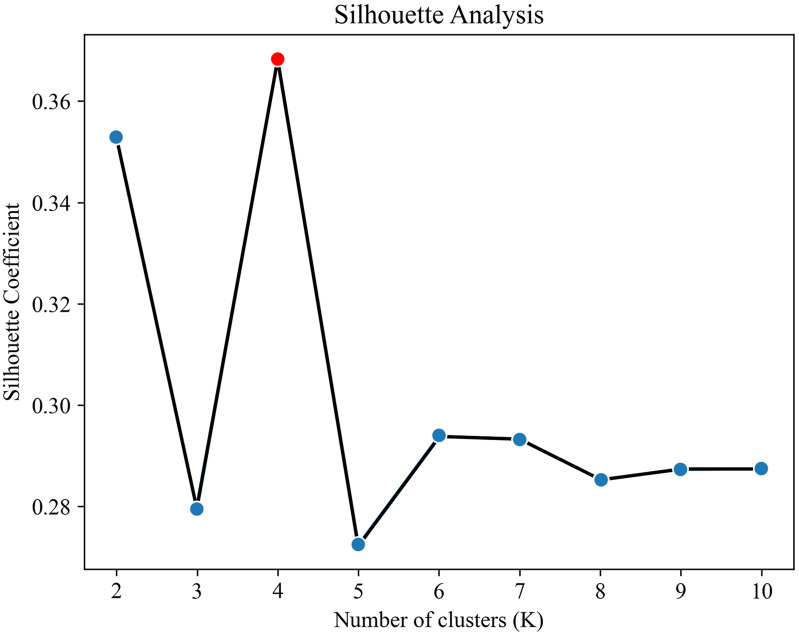
Silhouette coefficient plot based on NPJ model results.

**Fig 4:**
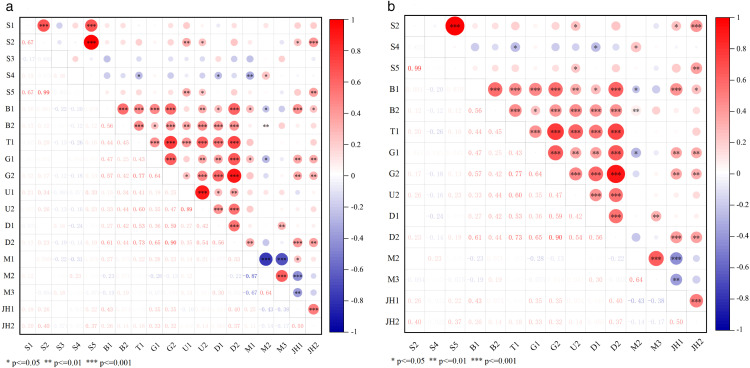
Correlation analysis result. (a) All indicators. (b) Selection indicators.

**Fig 5:**
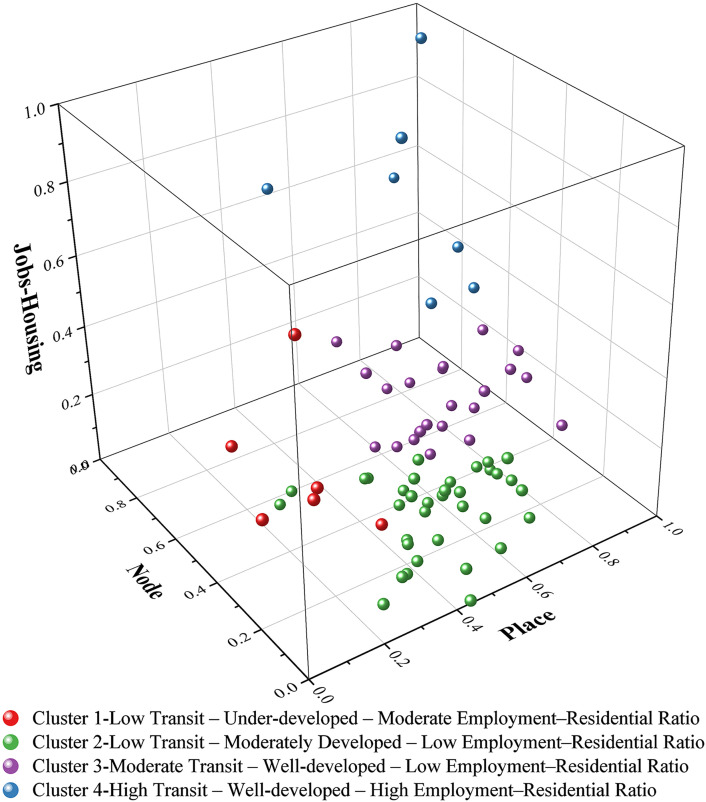
Scatter plots of clusters according to node, place, and jobs-housing value. (a) Cluster1: Low Transit, Under-developed, Moderate Employment-Residential Ratio. (b) Cluster 2: Low Transit, Moderately Developed, Low Employment-Residential Ratio. (c) Cluster 3: Moderate Transit, Well-developed, Low Employment-Residential Ratio. (d) Cluster4: High Transit, Well-developed, High Employment-Residential Ratio.

**Fig 6:**
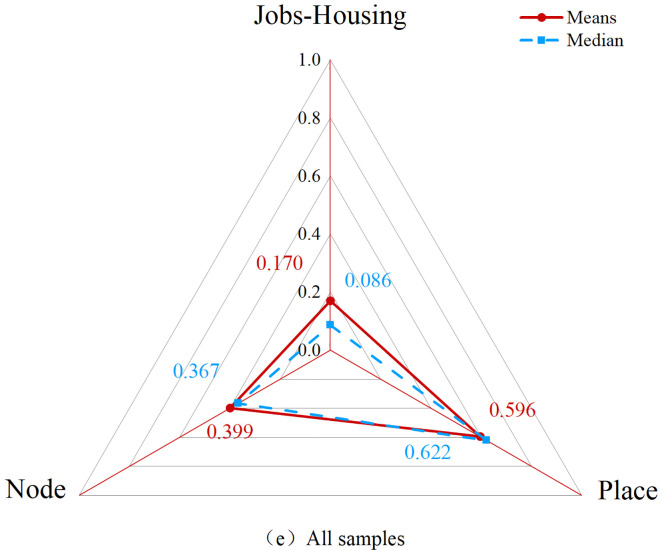
Radar plots of the node, place, and jobs-housing value of all samples.

**Fig 7:**
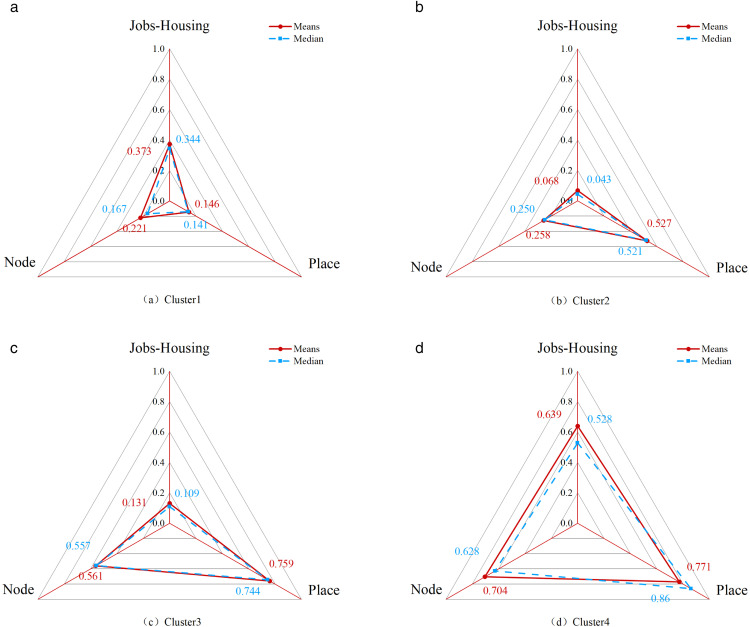
Radar plots of the node, place, and jobs-housing value in four clusters.

**Fig 8:**
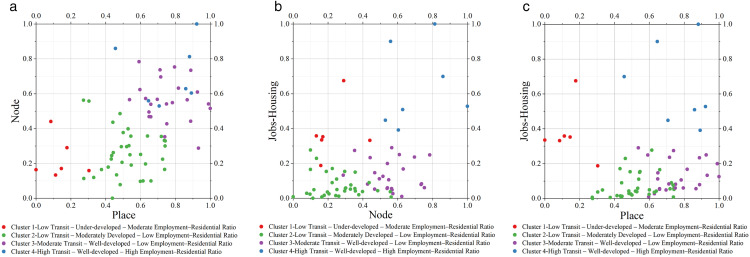
Scatter plots of clusters according to node, place, and jobs-housing value. (a) 2D scatter plot- Node—Place. (b) 2D scatter plot- Node—Jobs-Housing. (c) 2D scatter plot- Place— Jobs-Housing.

## 4 Methods

### 4.1 Formulation of model indicators

To construct the NPJ model, it is first necessary to establish the indicators for its three dimensions, NPJ-housing, with the calculation and data collection processes for each detailed in Table 1.

The indicators for the Node and Place dimensions are derived from those used in previous NP models [5,21,22,26,45,46,47], while also incorporating operational indicators for land use and traffic supply considered during the formulation of regulatory detailed planning, which ultimately determined the final indicator sets. Regulatory detailed planning serves as the statutory basis for land use in China. It is formulated by housing and construction departments at both the national and municipal levels. All entities responsible for design and construction—including those engaged in road development, transportation infrastructure, and building construction—must adhere to the parameter requirements specified in the regulatory detailed planning. Derived from the directives of the urban master plan, this regulatory document prescribes land use attributes, development intensity, building height limits, development density, greening coverage rate, and other relevant criteria for each plot. It also defines the construction standards for roads, such as the classification of roads, the number of lanes, the positioning of underground pipelines, and the surrounding greening coverage rates. As a rigid regulatory instrument, it plays a critical role in guiding and constraining urban construction and development management in China [71]. The Node dimension comprises eight indicators, categorized into three groups: subway supply capacity indicators (S), including betweenness centrality (S1), number of directions (S2), departure interval (S3), subway operating time (S4), and number of compartments (S5); bus supply capacity indicators (B), consisting of the number of bus stops (B1) and bus operating time (B2); and a private car supply capacity indicator (T), represented by parking lot density (T1). The indicator for the number of shared bicycles was excluded, as the average single-ride distance is approximately 1,000 meters [72], and within station areas, shared bicycles are typically used in combination with public transportation [73]. Moreover, in the context of this study, traffic supply capacity refers to the provision that supports inter-district commuting, and regulatory detailed planning generally does not stipulate the specific allocation of shared bicycles. The Place dimension includes nine indicators, organized into four categories: land development scale indicators (G), encompassing floor area ratio (G1) and population size (G2); urban construction form indicators (U), including total road length (U1) and number of intersections (U2); land development density indicators (D), consisting of building density (D1) and POI density (D2); and construction and development diversity indicators (M), which comprise land use mixture (M1), land use entropy (M2), and building type entropy (M3).

For the Jobs-housing dimension, we initially adopted the definition of the residential-employment ratio proposed by Horner. However, given that directly controlling the number of employment positions and housing units in land use planning is generally impractical, we modified this metric to better suit the study’s objectives. Consequently, two indicators were developed to represent the Jobs-housing dimension: the ratio of the floor area of employment buildings to that of residential buildings (JH1) and the ratio of the employment population to the residential population (JH2).

### 4.2 Selection of model indicators

Pearson correlation analysis was employed to identify the most effective indicators across the three dimensions [74]. Indicators showing no correlation with any variables from the other two dimensions were excluded, ensuring that each aspect within every dimension retained at least one relevant indicator. For cases in which the correlation coefficient between two indicators within the same aspect of the same dimension was ≥ 0.9 (*p *≤ 0.05), we further examined their correlations with indicators from the other dimensions and retained those demonstrating stronger interdimensional relationships.

### 4.3 Calculation methods for Node value, Place value, and Jobs-Housing value

This section synthesizes multiple indicators within each dimension, NPJ-housing, into a single dimensional value to support the visualization analysis requirements of the NP model [47,75]. The process addresses two primary challenges: the variation in measurement scales across indicators, resolved through min-max normalization, and the differences in their relative importance, addressed through weight allocation using the CRITIC method [76]. Unlike dimensionality reduction techniques, this approach preserves all original indicator information while enabling comparability of dimension values via weighted aggregation [77]. Specifically, min-max scaling normalizes each indicator by linearly mapping its values to the [0,1] range, thereby eliminating inconsistencies in units and value ranges across the original dataset. This normalization provides a standardized basis for subsequent weight allocation and the synthesis of dimensional values, with the min-max scaling formula expressed as follows:


$$X_{scaled}=\frac{X-X_{min}}{X_{max}-X_{min}}\;Positive$$

$$X_{scaled}=\frac{X_{max}-X}{X_{max}-X_{min}}\;Negative$$

Where $X_{scaled}$ represents the result of normalization, $X_{min}$ represents the minimum value within indicator X, $X_{max}$ represents the maximum value within indicator X.

After normalizing each indicator, weights are assigned using the CRITIC (Criteria Importance Through Intercriteria Correlation) method. Common approaches for determining the weights of indicators within a dimension include the analytic hierarchy process (AHP), the entropy weight method, and CRITIC. The AHP method relies on expert evaluations to establish a priority hierarchy of indicators within a dimension, followed by pairwise comparisons to derive their respective weights [46,26]. While effective for studies with a small number of indicators and limited data, AHP is highly dependent on subjective judgment and expert experience [78]. The entropy weight method determines weights based on the degree of dispersion in the data for each indicator [79], assigning higher weights to indicators with greater variability. However, it fails to account for correlations between indicators, which can result in disproportionately high weights for closely related variables [80,81]. In this study, data were collected from 72 subway station areas, covering 19 indicators and hundreds of thousands of individual data points, thereby meeting the requirements for applying the CRITIC method. CRITIC evaluates both the dispersion and intercorrelation of indicators: it calculates the standard deviation of each dataset to measure dispersion, identifies indicators with high correlation coefficients using Pearson correlation, and integrates these results to assign final weights. The formula for calculating CRITIC weights is as follows:


$$\begin{array}{c} C_{k}=\sigma_{k}\;\times \;\sum \left(\text{1}-r_{ki}\right) \end{array}$$

$$\begin{array}{c} w_{k}=\frac{C_{k}}{\sum_{k=\text{1}}^{n}C_{k}} \end{array}$$

$$S_{n,p,j}=\sum X_{nk,pk,dk}\times W_{nk,pk,dk}$$

where $C_{k}$ denotes the information content of indicator *k*; $\sigma_{k}$ is the standard deviation of all samples for indicator *k*, reflecting the degree of dispersion or variability in the data (where a larger standard deviation indicates greater variability); $\;r_{ki}$ represents the Spearman correlation coefficient between indicators *k* and *i* within the same dimension, indicating the strength of their linear relationship, or “conflict” (a higher correlation coefficient implies lower conflict and greater substitutability between the indicators); and $w_{k}$ is the assigned weight of indicator *k*. Once all indicator weights have been determined, the following formula is applied to compute the dimensional values:

where $S_{n,p,j}$ denotes the values for the NPJ-housing dimensions, respectively; $X_{nk,pk,dk}$ represents the normalized value of indicator *k* within the NPJ-housing dimensions, respectively; and $W_{nk,pk,dk}$ corresponds to the weight of indicator *k* within each of these dimensions.

The CRITIC weighting method and the min-max scaling normalization approach described above have also been employed in previous NP model studies examining the performance matching of station areas [82,83].

### 4.4 Calculation methods for TODness

TODness, introduced by scholars such as Su et al. (2021) and Yang et al. (2022), is an index used to quantify the degree of TOD development within station areas [5,75]. It is calculated using the Node value, the Place value, and the variance between these two measures. Prior NP model studies emphasize that, in implementing the TOD mode, it is necessary to evaluate both the construction intensity of TOD, reflected in the magnitudes of the Node and Place values, and the degree of coordination between transportation and place development, represented by the dispersion between these values [2,5]. A smaller variance between the Node and Place values indicates lower dispersion, implying a more balanced relationship between transportation infrastructure and place development. The formula for calculating TODness is therefore expressed as follows:


$${TODness}_{n}={(S}_{n}+S_{p})\times (\text{1}-v_{n})$$

where $S_{n}$ represents the Node value of station area *n*, $S_{p}$ denotes the Place value of station area *n*, and $v_{n}$ refers to the variance between the Node and Place values for station area *n*.

### 4.5 Classification methods for station area samples

After calculating the NPJ-housing values across the three dimensions, this study classifies the samples according to their dimensional characteristics to identify numerical differences among categories and analyze the spatial distribution patterns of each category within the urban context. In prior NP model research, data-driven classification methods are frequently employed, with three main approaches being Hierarchical Clustering [84,85], Two-step Clustering [26], and K-Means Clustering [47,42]. For this study, K-Means clustering was selected for several reasons: (1) Data suitability, K-Means is designed for numerical datasets, and the three-dimensional indicators (Node value, Place value, and Jobs-housing value) have already undergone min-max normalization and weighting, thereby meeting the algorithm’s requirement for data consistency; (2) Clustering shape assumption, Preliminary analysis suggests that the data exhibit an approximately spherical cluster distribution, aligning with K-Means’ convex clustering assumption; and (3) Sample size consideration, With 72 samples consisting entirely of numeric variables, hierarchical clustering’s dendrogram analysis and the mixed-data handling capabilities of two-step clustering are unnecessary, making K-Means the more appropriate choice.

The K-Means clustering method requires specifying the number of clusters, *K*. To determine the optimal value, this study employs the silhouette coefficient, a metric that simultaneously evaluates intra-cluster compactness and inter-cluster separation to assess clustering performance [86]. A silhouette coefficient value closer to 1 indicates superior clustering quality. As shown in Fig 3, the silhouette coefficients for all samples, calculated based on the NPJ-housing values, reach their maximum when *K* is set to 4. Accordingly, we set *K* = 4, classifying all samples into four distinct categories.

### 4.6 The role of TODness in Jobs-housing value

In this study, although cross-sectional data were collected from 72 station areas at the end of 2024, rather than time-series data from a single station area, these samples display varying degrees of TOD development and progress, as well as differences in employment-residential ratios. Moreover, all samples are located within the same construction context and economic region. Consequently, regression analysis is employed to examine the effect of TODness values on the Jobs-housing value for these 72 samples.

During the data exploration phase, it was observed that the relationship between TODness and the Jobs-housing value might not follow a linear pattern. We hypothesize the presence of an inflection point in the effect of TODness on the Jobs-housing value. To test this assumption, polynomial regression analysis was applied [87], with the regression equation expressed as follows:


$${TODness}_{ci}={TODness}_{i}+{TODness}_{avg}$$

$$\text{Jobs}-\text{housing value}=\beta_{\text{0}}+\beta_{\text{1}}{TODness}_{c}+\beta_{\text{2}}{TODness}_{c}^{\text{2}}+\varepsilon$$

In this equation, ${TODness}_{i}$ represents the TODness value of the station area indexed by *i*, ${TODness}_{avg}$ denotes the average TODness value across all station area samples, and ${TODness}_{ci}$ is the centered TODness value for station area iii. ${TODness}_{c}$ refers to the centered TODness variable, while ${TODness}_{c}$ denotes its squared term. The coefficients $\beta_{\text{0}}$, $\beta_{\text{1}}$, and $\beta_{\text{2}}$ are the regression parameters, and ∊\epsilon∊ represents the error term.

Because of the inherently strong linear relationship between independent variables and their higher-order terms in a polynomial regression model, variance inflation factor (VIF) values can become excessively high. To mitigate this issue and preserve the model’s generalization ability, mean-centering was applied to the independent variables by subtracting the overall sample mean from each value, thereby generating ${TODness}_{c}$ and ${TODness}_{c}^{\text{2}}$ were subsequently included in the regression. The validity of the regression model was evaluated using multiple criteria: analysis of variance (ANOVA, *p *≤ 0.05), VIF test (VIF < 5), Durbin-Watson (D-W) statistic (D-W value ∈ [1,3]), and standardized residual scatter plots (with residuals approximately distributed within ±3 and forming a rectangular pattern) [88].

## 5 Results and discussion

### 5.1 Indicator selection and correlation analysis results for the NPJ model

Fig 4a presents the correlation results for all 19 selected indicators, while Fig 4b shows the indicators retained after correlation analysis, resulting in a total of 15 indicators with 4 ineffective ones removed. In the Node dimension, S1 (betweenness centrality of the subway station) and S3 (departure interval) were excluded due to their lack of correlation with indicators in other dimensions. In the Place dimension, U1 (total road length) and M1 (land use mixture) were removed. U1 was excluded because it exhibited a high correlation with U2 correlation coefficient ≥ 0.886, *p *≤ 0.01), and U2 demonstrated stronger correlations with indicators across dimensions. Similarly, M1 was excluded due to its high correlation with M2 (correlation coefficient ≥ 0.873, *p *≤ 0.01), with M2 showing stronger cross-dimensional correlations.

Using the statistical values of the 14 effective indicators, we calculated the Node values, Place values, and Jobs-housing values for the 72 samples following the methodology outlined in Section 4.3. The TODness values for these station areas were also computed, and a correlation analysis was performed among the four values. Table 2 summarizes the results. The correlation coefficient between Node value and Place value is 0.515, while that between Node value and TODness is 0.889. Similarly, the correlation coefficient between Place value and TODness is 0.848. As this study focuses on the relationship between Jobs-housing value and the other variables, it is notable that the correlation coefficient between Jobs-housing value and Node value is 0.362, and between Jobs-housing value and TODness is 0.241. No significant correlation was observed between Jobs-housing value and Place value.

**Table 2:** Correlation analysis of node value, place value, TODness, and Jobs-Housing value (N = 72).

Variables for correlation analysis	Result	
	Pearson Correlation	P-value (Two-tailed)
Node value – Place value	0.515**	<0.001
Node value –TODness	0.889**	<0.001
Place value –TODness	0.848**	<0.001
Jobs-Housing value –Node value	0.362**	0.002
Jobs-Housing value –Place value	0.044	0.716
Jobs-Housing value –TODness	0.241*	0.041

** Correlation is signification at the 0.01 level; * Correlation is signification at the 0.05 level.

There is a significant linear correlation among Node value, Place value, and TODness, indicating a strong positive relationship between regional transportation supply and land use around stations under the TOD development mode. This result is consistent with previous studies conducted in various cities and regions near TOD stations [24,22]. Since TODness is calculated as the inverse of the variance between Node value and Place value, it naturally maintains a significant positive correlation with both [5,26]. However, contrary to expectations, the Jobs-housing value exhibits only a linear correlation with traffic supply and the TOD development index, while showing no correlation with land use. To further investigate this, we examined the correlation between two indicators under the Jobs-housing dimension and seven indicators under the Place dimension. JH1 (employment-residential area ratio) is linearly correlated with five land use indicators: G1, G2, D2, M2, and M3. JH2 (employment-residential population ratio) shows linear correlations with three land use indicators: G1, G2, and D2. These results indicate that the employment-residential area ratio is significantly associated with land development scale, land development density, and construction and development diversity, with the latter exhibiting an inverse relationship. The employment-residential population ratio is positively correlated with land development intensity, but demonstrates no correlation with construction form, development density, or diversity.

In 2021, Shenzhen initiated efforts to achieve a balanced employment-residential ratio through land spatial planning [89]. National policy approval explicitly emphasized that, based on the optimization of urban density zones, strategies such as mixed land use (increasing land use entropy), vertical development (increasing building mixed entropy), and the enhancement of community service facilities (increasing POI density) should be implemented to promote employment-residence balance [89,90]. The correlation analysis in this study reveals three key findings: (1) The public traffic capacity—reflected in the number of subway directions (S2), the number of subway compartments (S5), and the density of bus stops (B1) — shows a positive correlation with both the employment population and the building development area in station zones, and is associated with a high employment-residential ratio. (2) Higher development intensity and density, measured by the floor area ratio of the station area (G1), population size of the station area (G2), and POI density of the station area (D2), also show a positive correlation with the employment population and the building development area, and are linked to a high employment-residential ratio. (3) Greater diversity in land development, reflected in land use entropy (M2) and building mixed entropy (M3), shows a negative correlation with the residential building development area and is associated with a low employment-residential ratio. That is to say, most of the planning methods (6 indicators) under the TOD mode promote the station area’s employment-residential ratio to tilt towards employment. Only the planning method of land construction and development diversity (2 indicators) inhibits this tilt. This confirms the research conclusion of Xingang Zhou et al. Furthermore, existing research shows that Shenzhen’s central urban area is dominated by tertiary industries such as office and commercial activities, and exhibits a much lower employment self-sufficiency rate compared to suburban areas dominated by secondary industries [91]. Workers in the tertiary sector often choose to live far from their workplaces to reduce housing costs, relying heavily on the city’s developed public transportation network after the mature implementation of TOD [91]. Based on the correlation analysis results of this study, we posit that in Density Zone 1 of Shenzhen, strategies such as mixed land use (increasing land use entropy) and three-dimensional development (increasing building mixed entropy) may serve as potential strategies aimed at curbing an excessive shift of the employment-residential ratio toward employment dominance in TOD station areas.

### 5.2 TOD station area cluster analysis based on the NPJ model

Fig 5 illustrates the clustering distribution of the 72 station samples within the NPJ model. The average Node value for all samples is 0.399 (median: 0.367), the average Place value is 0.596 (median: 0.622), and the average Jobs-Housing value is 0.170 (median: 0.086; Fig 6). The distribution analysis shows that both Node values and Place values follow a normal distribution, whereas Jobs-Housing values display positive skewness (S4 File for details).

Fig 7 presents a radar chart illustrating the mean Node value, Place value, and Jobs-Housing value across the four identified clusters. Cluster 1 comprises six station areas and records the lowest Node value (average: 0.221, median: 0.141) and Place value (average: 0.146, median: 0.141) among all groups, while exhibiting the second-highest Jobs-Housing value (average: 0.373, median: 0.344). Cluster 2 includes 35 station areas, characterized by a low Node value (average: 0.258, median: 0.250), a moderate Place value (average: 0.527, median: 0.521), and the lowest Jobs-Housing value (average: 0.068, median: 0.043). Cluster 3 consists of 24 station areas with a moderate Node value (average: 0.561, median: 0.557), a high Place value (average: 0.759, median: 0.744), and a relatively low Jobs-Housing value (average: 0.131, median: 0.109). Cluster 4 contains seven station areas and demonstrates the highest scores across all three dimensions: Node value (average: 0.704, median: 0.628), Place value (average: 0.771, median: 0.860), and Jobs-Housing value (average: 0.639, median: 0.528).

Based on the relative characteristics of each cluster, we assigned descriptive labels as follows: Cluster 1 – Low Transit, Under-developed, Moderate Employment-Residential Ratio; Cluster 2 – Low Transit, Moderately Developed, Low Employment-Residential Ratio; Cluster 3 – Moderate Transit, Well-developed, Low Employment-Residential Ratio; and Cluster 4 – High Transit, Well-developed, High Employment-Residential Ratio. This nomenclature aligns with the typology proposed by Wu et al. (2024) in their extension of the NPV (Vitality) model [92]. Furthermore, the classification approach is consistent with typological frameworks used in previous employment-residential ratio studies from a land use perspective [93].

Incorporating Jobs-Housing as a third dimension effectively enhances the original NP model, resulting in the NPJ model. Analysis of the average NPJ-Housing values across the four clusters shows that both Node and Place values consistently increase from Cluster 1 to Cluster 4. In contrast, the Jobs-Housing value declines from Cluster 1 to Cluster 2, then rises steadily from Cluster 2 to Cluster 4. This pattern suggests that, while Jobs-Housing values exhibit a linear correlation with TODness, the degree of TOD development in station areas may influence the employment-residential ratio in a non-linear manner.

### 5.3 Comparison of categories based on the NPJ Model and types based on the NP Model

**Fig 9:**
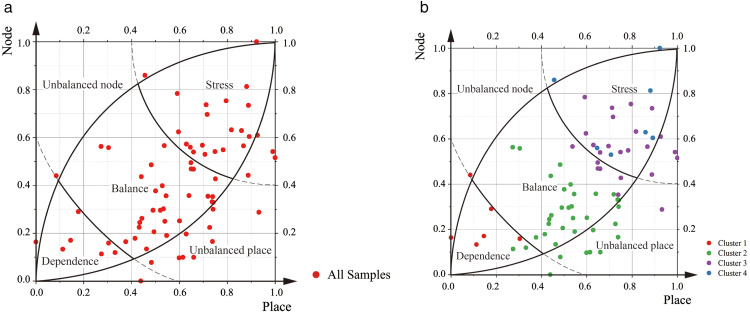
Revised Node–Place spatial typology model. (a) unascertained cluster. (b) Clustered.

Fig 8 presents the distribution of all samples across the three pairwise dimensions of the NPJ model: NP, Node-Jobs-Housing, and Place-Jobs-Housing. Fig 9a illustrates the classification and spatial distribution of all samples within the original NP model, while Fig 9b depicts the distribution of the four NPJ clusters in the NP quadrant. The detailed classification of all samples within the four NPJ clusters, along with their allocation to the five typologies of the NP model, is provided in S2 Table.

In Cluster 1, 67% (4 of 6 samples) are classified as Dependence in the NP model, characterized by low transportation supply and low land use, while the remaining 33% (2 samples) fall under the Unbalanced Node type, with high transportation supply but low land use. In Cluster 2, 6% (2 of 35 samples) are Dependence, 69% (24 samples) are Balance, moderate traffic supply and land use in equilibrium, and 26% (9 samples) are Unbalanced Place, with low transportation supply but high land use. Cluster 3 consists of 21% (5 of 24 samples) Unbalanced Place, 25% (6 samples) Balance, and 54% (13 samples) Stress, the latter reflecting high transportation supply and high land use in balance. Cluster 4 includes 29% (2 of 7 samples) Unbalanced Node and 71% (5 samples) Stress.

In expanding the third dimension of the NP model, numerous studies have explored classification approaches based on newly developed models [42,26,5]; however, limited attention has been given to examining the correspondence between the clusters of these new models and the original NP model types. The four clusters identified through the incorporation of the Jobs-Housing dimension exhibit varying degrees of alignment with the five established NP model types. Specifically, the “Low Transit, Under-developed, Moderate Employment-Residential Ratio” cluster corresponds to the Dependence and Unbalanced Node types, encompassing samples with Node values between 0.1 and 0.5 and Place values between 0 and 0.3, where employment-to-residential building area ratios and employment-to-residential population ratios are moderate among all samples. The “Low Transit, Moderately Developed, Low Employment-Residential Ratio” cluster is predominantly derived from the Balance type, with some representation from the Dependence and Unbalanced Place types, characterized by Node values between 0 and 0.6 and Place values between 0.3 and 0.8, and exhibiting the lowest employment-to-residential ratios, indicating a substantial surplus of residential building area and population compared to employment facilities and workforce. The “Moderate Transit, Well-developed, Low Employment-Residential Ratio” cluster, consisting of samples evenly distributed between the Stress, Unbalanced Place, and Balance types, includes cases with Node values between 0.3 and 0.8 and Place values between 0.5 and 1.0, where employment-to-residential ratios remain relatively low. Lastly, the “High Transit, Well-developed, High Employment-Residential Ratio” cluster primarily originates from the Stress type, with a minor contribution from the Unbalanced Node type, covering samples with Node values between 0.5 and 1.0 and Place values between 0.5 and 1.0, and showing comparatively high employment-to-residential ratios, where employment building areas and populations exceed their residential counterparts.

In situations where employment-residential ratio data for a station area is scarce or the development ratio remains undefined during the detailed planning stage, the observed values or planning targets for traffic supply (Node) and land use (Place) can be matched to the corresponding sample categories, enabling the estimation of the Jobs-Housing value and providing a preliminary forecast of both the commercial-to-residential building area ratio and the working-to-residential population ratio within the station area.

### 5.4 Land use characteristics and spatial distribution of the four clusters

**Fig 10:**
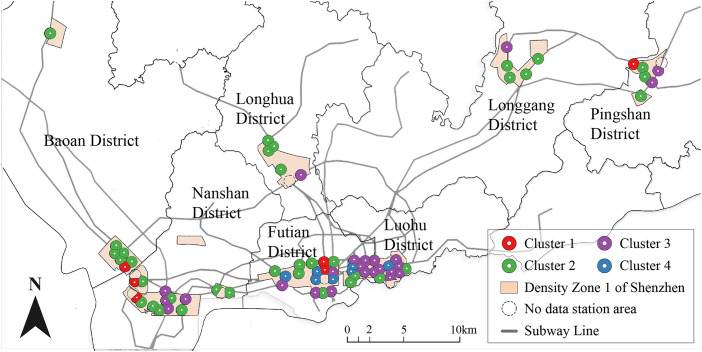
Spatial typology of station clusters in Density Zone 1 in Shenzhen. Base map data from OpenStreetMap. The figure is a simplified representation for illustrative purposes.

Fig 10 depicts the spatial distribution of the four clusters in the NPJ model within the sampling area (Density Zone 1 of Shenzhen), encompassing seven natural planning zones arranged from east to west, while Table 3 summarizes the corresponding distribution of sample quantities across these districts.

**Table 3:** Distribution of sample quantities for the four clusters in the seven natural planning districts.

Natural Planning Area	Pingshan District				Longgang District				Luohu District				Longhua District				Futian District				Nanshan District				Baoan District			
Sample Size	6				5				10				5				26				14				7			
Cluster	1	2	3	4	1	2	3	4	1	2	3	4	1	2	3	4	1	2	3	4	1	2	3	4	1	2	3	4
Sample Count	1	3	2	0	0	4	1	0	0	2	7	1	0	4	1	0	2	10	9	6	2	8	4	0	1	6	0	0

Cluster 1 consists of six samples: Pingshan Station in Pingshan District; Guiwan and Menghai station areas in Nanshan District; Linhai station area in Bao’an District; and Civic Center and Youth Palace station areas in Futian District. Based on the territorial spatial planning and regulatory detailed plans of Pingshan, Nanshan, and Bao’an Districts [89,94–97], combined with land use data and Google Maps [98], the first four station areas are characterized by low construction density due to the presence of extensive parks and green spaces. In contrast, the last two station areas host significant urban public buildings, such as the Shenzhen Civic Center and Shenzhen Youth Palace, along with large urban squares, which also result in relatively low construction density [94,98,99] (Futian District regulatory detailed planning, Google Maps of Futian Central City). These areas are typically located near urban natural landscapes or concentrated administrative centers [98].

Cluster 2 comprises 35 samples distributed across the seven natural planning districts. According to Fig 10, these samples are primarily situated on the periphery of the urban core areas (Density Zone 1) in Luohu and Futian Districts, surrounding Cluster 3 and Cluster 4 locations. In these station areas, land is mainly developed for residential buildings, as indicated by the territorial spatial planning and regulatory detailed plans of Luohu and Futian Districts [89,94,99] and Google Maps [98]. In Pingshan, Longgang District, Longhua District, Nanshan District, and Bao’an District, Cluster 2 samples exhibit two distinct scenarios [94,98]: one scenario involves station areas predominantly developed for residential buildings, such as Baohua, Bao’an, Hongshan, Jinlong, and Longcheng Park station areas, while the other includes areas with significant urban public buildings, where residential and public facility developments each occupy nearly half of the land, exemplified by Dayun Center station area (which includes a sports venue) and Bao’an Central station area (housing the Bao’an District Government and Cultural Arts Center). In developed central urban areas, such as Luohu District, Futian District, and Nanshan District, these types of station areas are often located at the periphery of the city center. In the central urban areas of the city’s sub-centers within these districts, such station areas are generally located in the cores of the central urban zones.

Cluster 3 comprises 24 samples distributed across six natural planning districts, excluding Bao’an District. Based on the territorial spatial planning and regulatory detailed plans of Luohu District, Futian District, Nanshan District, Pingshan District, Longgang District, and Longhua District [94–96,99–102], along with land use data and Google Maps [98], residential and office building developments each occupy roughly half of the construction land in these station areas. These areas are scattered throughout the urban center, exhibiting no distinct spatial distribution patterns.

Cluster 4 comprises seven samples, exclusively located within Density Zone 1 of Luohu and Futian Districts. Laojie station is situated in Luohu District, while the remaining six stations are in Futian District. According to Shenzhen’s master planning and urban development history [89], the central urban areas of Luohu District and Futian District, coinciding with Density Zone 1, are the earliest and most developed regions of the city. Among these, Laojie, Huaqiangbei, and Shopping Park station areas are prominent commercial hubs, whereas Chegongmiao, Gangxia, Futian Station, and Huaqiang Road station areas serve as major office clusters. Except for Huaqiang Road station, all six function as multi-line transfer hubs, suggesting that subway stations in well-developed urban centers, dominated by commercial and office developments, correspond to the “High Transit, Well-developed, High Employment-Residential Ratio” category.”

Based on the analysis of land use and spatial distribution characteristics of the four station area clusters, Table 4 was compiled to summarize the findings. The study adopts a balance index, defined as the ratio of employment positions to housing units within a given area, which is considered balanced when its value ranges between 0.8 and 1.2 [8]. Comparing the average employment-to-residential building area across the four clusters, Clusters 2 and 3 fall within this balanced range, whereas Cluster 1 (underdeveloped land) and Cluster 4 (high development intensity) are classified as unbalanced, both exhibiting larger employment building areas and higher employment populations. Although Cluster 3 meets the balance index criteria, it still demonstrates a greater employment building area and more employed individuals relative to residents. Collectively, Clusters 1, 3, and 4 comprise 37 samples (51.4% of the total), indicating a stronger tendency for employment-related building development and workforce concentration. This contrasts with the study by Yang and Chang (2025), which examined light rail areas in South Korea and highlighted an imbalance favoring residential development around suburban city center stations due to enhanced transport convenience [50]. In contrast, our analysis of Shenzhen’s central urban density zone indicates that only half of the station areas (35 samples in Cluster 2) show a slight preference for residential development, while the remaining areas are more attractive for employment building and related workforce growth.

**Table 4:** Analysis of the characteristics of the four clusters.

Cluster	Characteristics of the three dimensions	Land use characteristics within station areas	Spatial distribution in the city	Employment building area/ residential building area (avg.)	Employment population/ residential population (avg.)	Jobs-Housing value(avg.)	Jobs-housing characteristics
1	Low Transit, Under-developed, Moderate Employment-Residential Ratio	1 The station area features large parks and green spaces. 2 The station area includes urban public facilities and city squares, which together occupy more than half of the land in the station area.	1 Adjacent to natural landscapes of neighboring cities (such as coastal areas and foothills). 2 Located in the urban administrative center.	4.878	2.361	0.373	Employment dominant station area with an imbalance between jobs and housing, characterized by low land development intensity.
2	Low Transit, Moderately Developed, Low Employment-Residential Ratio	1 A significant portion of the station area land is used for residential development. 2 A large amount of the station area land is used for both residential development and urban public facilities, with each accounting for nearly half of the construction land in the station area.	1 In well-developed urban areas, they are often found at the periphery of the central urban district. 2 In rapidly developing urban areas, they are predominantly located in the core of the central urban district.	0.872	0.602	0.068	Residentially biased station area with jobs-housing balance.
3	Moderate Transit, Well-developed, Low Employment-Residential Ratio	The land in the station area is allocated for both residential development and office and commercial development, with each accounting for nearly half of the total construction land in the station area.	Randomly distributed within the urban central district.	1.189	1.205	0.131	Employment biased station area with jobs-housing balance.
4	High Transit, Well-developed, High Employment-Residential Ratio	The land in the station area is allocated for both residential development and office and commercial development, with each accounting for nearly half of the total construction land in the station area.	Predominantly located in renowned commercial street areas and clusters of office developments within the city.	6.401	4.852	0.639	Employment dominant station area with an imbalance between jobs and housing, characterized by high land development intensity.

Analyzing the land use characteristics and spatial distribution of station areas can assist urban planners in strategically positioning these zones. In urban center regions, the two jobs-housing imbalance clusters (Cluster 1 and Cluster 4) are predominantly driven by employment development, allowing for rapid classification and decision-making regarding land use proportions. Cluster 1 station areas, located near urban natural landscapes (such as coastlines or foothills) and administrative centers, typically allocate large portions of land to green spaces, plazas, and public facilities, making it challenging to enhance development intensity and diversity, with commercial, office, and urban public facilities dominating the limited buildable land. Cluster 4 station areas, situated in major commercial districts and office clusters, generally dedicate approximately half of their construction land to commercial and office buildings and the other half to residential development, resulting in an employment-residential ratio skewed toward employment. In contrast, Clusters 2 and 3 display balanced jobs-housing area ratios without specific topographical or locational constraints. Cluster 2 areas primarily feature extensive residential development or a near-equal distribution between residential and urban public facilities, such as sports stadiums, art galleries, and government service halls. Cluster 3 areas allocate roughly half of the land to commercial and office buildings and half to residential use, typically as single-line subway stops. These clusters can generally adopt the land planning approaches of Shenzhen’s Density Zone 1, as their jobs-housing imbalance is typically not severe. It is noteworthy, however, that some Cluster 3 station areas share key characteristics with Cluster 4, such as evolving into multi-line transfer hubs and catalyzing new office or commercial districts, suggesting a potential developmental trajectory for them. For these Cluster 3 station areas with development potential, consideration should be given to the early implementation of mixed land use (increasing land use entropy) and three-dimensional development (enhancing building mixed entropy), which could help prevent the employment-residential ratio from becoming excessively skewed toward employment during the development process.

### 5.5 Interaction between TOD development degree and the employment-residential ratio in station areas

The TOD index (TODness) quantifies the degree of coordinated development between traffic supply (Node) and land use (Place) in station areas [5,12] and serves as a key metric for assessing the degree of TOD development. Its measurement encompasses two components: construction intensity, evaluated as the sum of Node and Place values, and construction equilibrium, assessed by the variance between Node and Place values, where smaller variance indicates a more balanced transportation-land development [2,5]. TODness reflects the overall construction degree of Node and Place within station areas, providing the foundational conditions for establishing jobs-housing relationships. Theoretically, TOD development is considered a key factor influencing the employment-residential ratio by altering transportation accessibility and shaping the spatial distribution of employment and residential areas in station zones. Concurrently, changes in jobs-housing demand may also exert a regulatory feedback effect on station-area transportation and land development [51]. However, this cross-sectional study primarily reveals associations between these variables, rather than establishing causal directions.

The review and logical inference in Section 2.3 suggest a correlation between TODness and the employment-residential ratio in station areas. Correlation analysis in Section 5.1 reveals a linear relationship between the Jobs-Housing value and TODness, and the analysis of mean values across three dimensions of various TOD clusters in Section 5.3 indicates the presence of a turning point in the employment-residential ratio as TODness varies. Therefore, linear and polynomial regression methods, as discussed in Section 4.5, are employed to explore the impact of TODness on the Jobs-Housing value, with TODness, ${TODness}_{c}$, and ${TODness}_{c}^{\text{2}}$ as independent variables and the Jobs-Housing value as the dependent variable for linear fitting.

**Fig 11:**
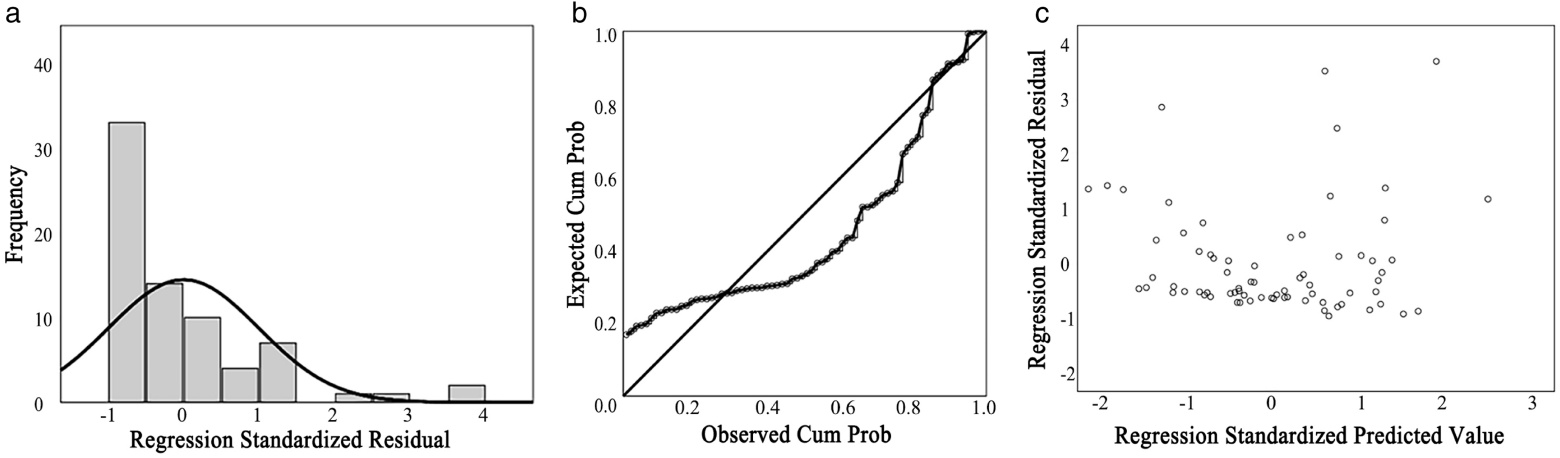
Plots of the results of the validation analysis of the linear regression model (all samples). (a) Histogram of standardized residuals. (b) Normal P-P plot of standardized residuals. (c) Scatterplot of standardized residuals.

First, a univariate linear regression analysis was conducted on the Jobs-Housing value (Table 5), resulting in the fitted equation: Jobs-Housing value = 0.043 + 0.241 × TODness. The significance tests, D-W statistic, and residual analyses (Table 5 and Fig 11) confirm that the linear regression results are statistically significant. The cross-sectional data reveal that higher TODness values are associated with a higher employment-residential ratio, characterized by larger employment-building areas and a greater working population. However, the model explains only 5.8% of the variance in the Jobs-Housing value.

**Table 5:** Regression results and test statistics for the independent variables on the dependent variables (all samples).

Model summary (R² = 0.058)				Diagnostic statistics		
	β	t	Sig.	ANOVA		D-W Value
Constant	0.043	0.648	0.519	F	Sig.	
TODness	0.241	2.079	0.041*	4.323	0.041*	1.845

Note: Dependent variable: Jobs-housing value; Independent variables: TODness.

* Correlation is signification at the 0.05 level.

**Fig 12:**
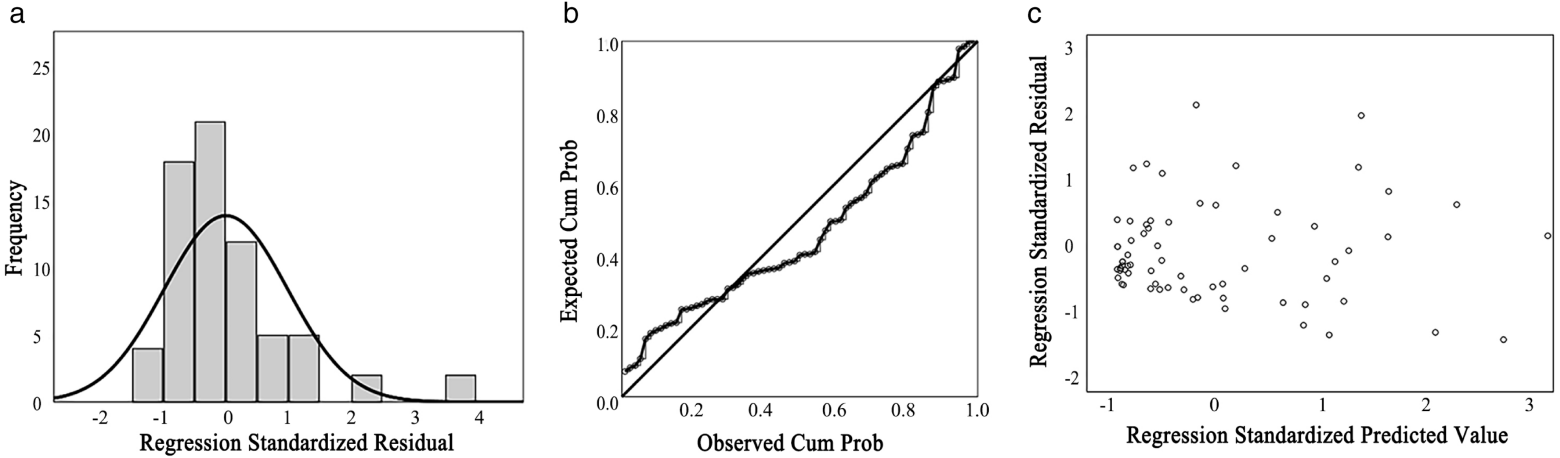
Plots of the results of the validation analysis of the linear regression model (all samples). (a) Histogram of standardized residuals. (b) Normal P-P plot of standardized residuals. (c) Scatterplot of standardized residuals.

A polynomial regression analysis was performed on the Jobs-Housing value (Table 6), yielding the fitted equation: Jobs-Housing value =$\text{0}.\text{1}\text{0}\text{6}+\text{0}.\text{1}\text{6}\text{2}\times {TODness}_{c}+\text{0}.\text{4}\text{0}\text{3}\times {TODness}_{c}^{\text{2}}$. The significance tests, D-W statistic, and residual analyses (Table 6 and Fig 12) indicate that the polynomial regression results are statistically significant. Compared with the linear model, the polynomial regression demonstrates improved explanatory power, accounting for 22.0% of the variance in the Jobs-Housing value.

**Table 6:** Regression results and test statistics for the independent variables on the dependent variables (all samples).

Model summary (R² = 0.220, Adjusted R² = 0.198)				Diagnostic statistics		
	β	t	Sig.	ANOVA		D-W Value
Constant	0.106	3.689	<0.001**	F	Sig.	
${TODness}_{c}$	0.162	1.685	0.097			
${TODness}_{c}^{\text{2}}$	0.403	3.785	<0.001**	9.737	<0.001**	1.915

Note: Dependent variable: Job-housing value; Independent variables: TODness.

** Correlation is signification at the 0.01 level.

**Fig 13:**
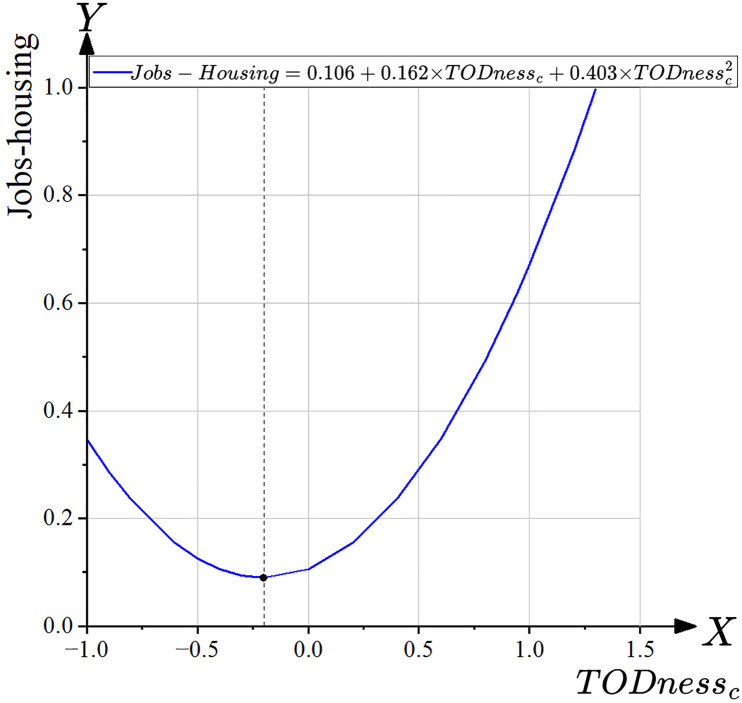
Polynomial linear fit of jobs-housing vs. ${TODness}_{c}$

**Fig 14:**
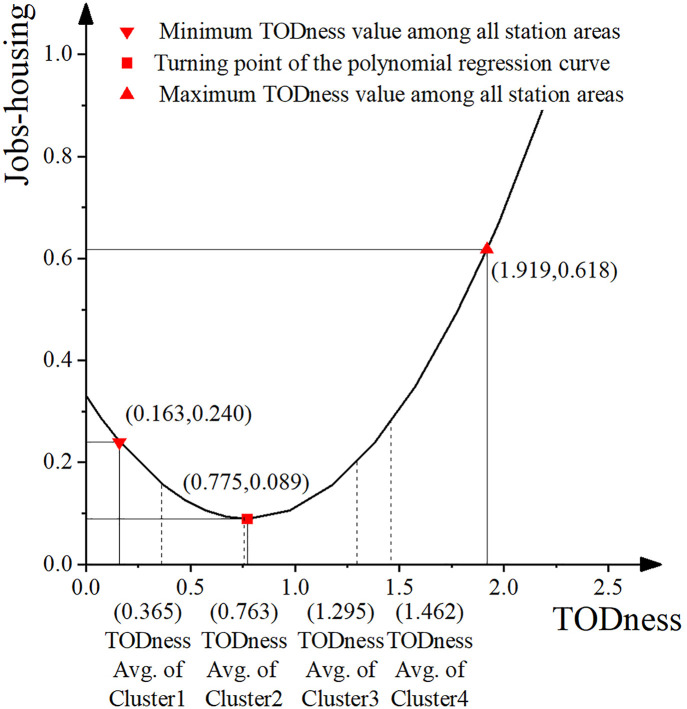
U-shaped effect of jobs-housing value on TODness (De-centered TODness).

The polynomial regression analysis suggests a U-shaped association between the level of TOD development and the employment-residential ratio, indicating the presence of an inflection point in this relationship (Fig 13). However, the centered variables, ${TODness}_{c}$ and ${TODness}_{c}^{\text{2}}$, lack practical explanatory or operational value for planning controls of traffic supply and land use. To address this, we identify the TODness value corresponding to the inflection point and examine the marginal effects of the nonlinear relationship. Using the formula from Section 4.5, the derived curve is mapped back into the TODness-Jobs-Housing quadrant, where the average ${TODness}_{avg}$ is 0.975, enabling determination of the inflection point and the upper and lower bounds of the U-shaped relationship (Fig 14). In this study, the minimum TODness among the samples is 0.163, the maximum is 1.919, and the inflection point occurs at 0.775, establishing that the U-shaped effect applies within the TODness range of 0.163 to 1.919. When TODness is below 0.775, the employment-residential ratio decreases as TODness increases, indicating larger residential building areas and populations; conversely, when TODness exceeds 0.775, the employment-residential ratio rises with TODness, reflecting larger employment building areas and populations. Previous studies on TOD station areas have not reached conclusive findings regarding the effect of TOD development degree on the employment-residential ratio.

From a land planning policy perspective, polynomial regression analysis further indicates that the development of the TOD mode in the later stage can lead to an employment-residential imbalance favoring employment, highlighting a key challenge in implementing TOD in high-density urban areas [103]. Anchored at transit nodes, the TOD mode drives station-area development, while enhanced traffic supply facilitates spatial decoupling between jobs and housing. Observations from central urban areas show that once a station area reaches the Cluster 2 stage, there is a persistent tilt toward employment in the employment-residential ratio (Fig 14). Although the polynomial regression sample consists of 72 cross-sectional cases rather than time series, reflecting varying TOD development degree and corresponding employment-residential differences, with some station areas still under development, significant trends emerge. For example, as discussed in Section 5.4, subway construction can produce multi-line transfer stations, catalyzing new office clusters or commercial districts, causing some areas to transition from Cluster 3 to Cluster 4. During the initial TOD implementation phase, particularly from Cluster 1 to Cluster 2, station areas exhibit a residential bias in the employment-residential ratio, reflecting their enhanced attractiveness for residential development and population concentration, suggesting that prioritizing residential land use aligns with early-stage TOD patterns. In the intermediate phase, transitioning from Cluster 2 to Cluster 3, station areas maintain balanced employment-residential ratios, where increased public transit provision, elevated station-area development intensity, and higher construction density collectively support employment-generating built environments and attract skilled labor; thus, urban planning strategies should emphasize commercial and office space development to optimize TOD outcomes. In advanced TOD stages, controlling excessive jobs-housing imbalance requires improving station-area employment self-sufficiency. Implementing mixed-use land policies and three-dimensional development strategies, through localized land-use adjustments, effectively increases land use entropy and building mixed entropy. Existing studies show that refined density and intensity controls can shape targeted TOD patterns [104]; however, our analysis indicates that interventions focused on development diversity and three-dimensional approaches are most effective during mid-to-late TOD phases.

## 6 Conclusion and limitations

This study successfully extends the NP model (a TOD typology tool) by developing the NPJ model through systematic integration of the jobs-housing dimension, empirically validating its effectiveness, and enhancing understanding of subway station areas as complex urban systems, while revealing systematic relationships among employment-residential ratios, traffic supply, and land use in high-density TOD development. The NPJ model and methodology developed in this study can be extended to other high-density cities and regions, particularly within densely populated central urban areas, enabling horizontal comparisons with the numerical results presented here. In addition, future research could replicate and expand upon our findings by examining how TOD development degree at station areas affect employment-residential ratios, as well as by testing the influence of individual indicators using alternative nonlinear models such as random forests.

In high-density urban areas, implementing the TOD development mode for subway station areas requires determining jobs-housing values based on TOD station area types, which in turn helps establish the ratios of built-up area and population for employment and housing. TOD station type classification can be assessed according to the surrounding land use characteristics and internal land planning (Table 4). This study identifies an imbalance in jobs-housing matching within the small-scale urban context of TOD subway station areas, occurring only in two types of areas: those with relatively low land development intensity (average Place value ≤ 0.146) and those with relatively high intensity (average Place value ≥ 0.771). In contrast, station areas with moderate land development intensity and diversity (average Place value between 0.527 and 0.759) maintain balanced employment-residential ratios, regardless of whether the area leans toward residential or commercial functions, as indicated by a balance index of 0.8–1.2. Furthermore, the TOD index (TODness) exhibits a U-shaped relationship with the employment-residential ratio in station areas. In Shenzhen’s highest-density construction zones, the inflection point occurs at a TODness value of 0.775, where early TOD development promotes greater growth of residential area and population, whereas in later stages, employment building area and population increase surpass those of residential development. Consequently, in advanced stages of TOD implementation in central urban station areas, an inherent occupational bias in employment-residential ratios emerges as an inevitable characteristic.

The findings of this study offer valuable guidance for establishing indicators and parameters in the regulatory detailed planning of urban development and for adjusting land construction interventions according to varying degrees of TOD development. In constructing subway systems in high-density urban areas, particularly central districts, coordinated planning and control should account for traffic supply, land use, and employment-residential ratios specific to each TOD station area type. During TOD implementation, employment-residential ratios exhibit distinct trends at different stages: in the early phases, residential land should be prioritized, while mid- and late-stage developments should focus on commercial and office spaces. This sequencing aligns with the inherent developmental patterns and effectiveness of TOD modes. Partial adjustments and mixing of land use within station areas aim to enhance development diversity, increasing land use entropy, and building mixed entropy. Such strategies are particularly effective during the mid-to-late stages of TOD implementation to prevent excessive skewing of the employment-residential ratio toward employment within the station area.

This study has several limitations. First, although we thoroughly examined subway station samples in Shenzhen’s highest-density development areas, the sample sizes for Cluster 1 and Cluster 4 are relatively small after clustering. Expanding the study to other cities or regions may introduce differences in construction contexts, such as land development intensity and industrial structures, which could limit the applicability of the NP static model. However, it is important to recognize that land development intensity benchmarks, industrial structures, and commuting patterns in other cities and regions differ from those in Shenzhen’s Density Zone 1. Consequently, the specific values derived from this study cannot be directly applied elsewhere. This includes the ranges of Node and Place values for the four categories, as well as average indicators such as employment building area/residential building area, employment population/residential population (avg.), employment-residential ratio (avg.), and the TODness inflection point of 0.775 in relation to the jobs-housing value. Other cities or regions may adopt the construction methodology of the NPJ model, together with the insights generated in this study regarding the relationship between employment-residential ratios, transportation supply, land-use patterns, and TOD indices, to determine appropriate numerical values for their own subway station area samples. As additional metro lines are developed in the study area, future research could increase sample sizes, leading to more robust conclusions. Second, the indicators used in constructing the NPJ model are commonly applied in urban planning for transportation, land use, and population; future studies may adjust these indicators according to different research objectives, potentially affecting numerical outcomes. Third, this research relies on cross-sectional data from December 2024. It should be noted that the 72 samples, despite varying in TOD development levels, do not constitute time-series data for individual stations. This implies that causal relationships between the dimensional indicators cannot be established, and the observed relationships represent merely correlations. Therefore, the inverse U-shaped relationship between TODness and the JH value in the polynomial regression, with an explanatory power of 22%, should be interpreted as a statistical association within the cross-sectional data. We posit that the explanatory power of the current model is partially constrained by this data structure; future research employing time-series data or quasi-experimental methods could potentially improve model fit and further infer the causal direction between variables. Finally, we mitigated the MAUP and station traffic supply drift by restricting the analysis area and standardizing station ranges; however, the actual impact zones of subway stations inevitably differ, necessitating correction through micro-level travel origin-destination (OD) data obtained from mobile.

## Supporting information

S1 File Mathematical derivation of the Node-Place (NP) model classification boundaries. This file provides the detailed mathematical principles and equations used to define the classification boundaries (Dependence, Stress, Unbalanced Node, Unbalanced Place) in the Node-Place model, addressing ambiguities in the original typology.
(DOCX)

S2 Table Comparison of NPJ categories and NP types of 72 subway station areas in Density Zone 1 of Shenzhen. This table provides a comparative analysis between the newly proposed Node-Place-Jobs-housing (NPJ) classifications and the traditional Node-Place (NP) typologies for the 72 studied subway station areas.
(DOC)

S3 Fig Spatial distribution and names of 72 subway station areas in Density Zone 1 of Shenzhen. Base map data from OpenStreetMap. The figure is a simplified representation for illustrative purposes. This figure depicts the geographical locations of the 72 subway station areas under investigation, with each station being geographically labeled, within Shenzhen’s Density Zone 1.
(TIF)

S4 File Normality tests and distribution histograms for key model variables. This file includes the complete results of normality tests (Kolmogorov-Smirnov and Shapiro-Wilk) and corresponding distribution histograms for Node values, Place values, Jobs-housing values, employment-residential area ratios, and employment-residential population ratios.
(DOC)
