# Supplementary material for: Construction of the Node—place—Jobs-housing model: Analysis of employment-residential ratio in subway station areas of Shenzhen, China’s highest construction density zone
Source: PLoS One. 2025 Dec 5;20(12):e0337576. doi: 10.1371/journal.pone.0337576 (PMC12680167; doi:10.1371/journal.pone.0337576)
Supplement: S1 File — This file provides the detailed mathematical principles and equations used to define the classification boundaries (Dependence, Stress, Unbalanced Node, Unbalanced Place) in the Node-Place model, addressing ambiguities in the original typology. (DOCX) [file pone.0337576.s001.docx]

In the study of TOD station area typology using the NP model, the construction principles of the NP model and the illustrations of the two-dimensional model are presented, along with five types identified: Balanced, Dependent, Pressured, Node Imbalance, and Place Imbalance [1,2]. However, within the NP model's two-dimensional coordinate system, two of the four arc lines that distinguish these five types are ambiguous (specifically, the lines distinguishing Stress from Balance and Balance from Dependence). Previous research has not conducted a comparative analysis of the expanded model types against the original types [3,4], leaving the issue of the two ambiguous classification boundaries in the NP model unaddressed. In this study, we attempt to locate the four boundary arc lines using equidistant circular arcs centered at key points. The mathematical principles for positioning each arc line are as follows:

| **Zone Type** | **Center** | **Radius** | **Arc Equation** |
| --- | --- | --- | --- |
| Dependence | (1, 1) | 1.16 | $(P-1)^{2}+(N-1)^{2}=1.16$ |
| Stress | (1, 1) | 0.6 | $(P-1)^{2}+(N-1)^{2}=0.36$ |
| Unbalanced Node | (1, 0) | 1 | $(P-1)^{2}+N^{2}=1$ |
| Unbalanced Place | (0, 1) | 1 | $P^{2}+(N-1)^{2}=1$ |

Variable definitions:

$N$: The standardized Node value, representing the diversity and intensity of traffic supply in the station area, as well as its accessibility.

$P$: The standardized Place value, representing the diversity and intensity of land-use activities within the station area.

1. Bertolini L. Station areas as nodes and places in urban networks: An analytical tool and alternative development strategies. In: Bruinsma F, Pels E, Rietveld P, Priemus H, van Wee B, editors. Railway Development: Impacts on Urban Dynamics. Heidelberg: Physica-Verlag HD; 2008. pp. 35–57. doi:10.1007/978-3-7908-1972-4_3

2. Bertolini L. Spatial Development Patterns and Public Transport: The Application of an Analytical Model in the Netherlands. Planning Practice and Research. 1999;14: 199–210. doi:10.1080/02697459915724

3. Lyu G, Bertolini L, Pfeffer K. Developing a TOD typology for Beijing metro station areas. Journal of Transport Geography. 2016;55: 40–50. doi:10.1016/j.jtrangeo.2016.07.002

4. Wu T, Li M, Gao L, Zhou Y. Integrating spatial vitality and node-place model to evaluate and classify metro station areas in Wuhan. Frontiers of Architectural Research. 2024 [cited 17 Sept 2024]. doi:10.1016/j.foar.2024.04.005
