# Supplementary material for: Construction of the Node—place—Jobs-housing model: Analysis of employment-residential ratio in subway station areas of Shenzhen, China’s highest construction density zone
Source: PLoS One. 2025 Dec 5;20(12):e0337576. doi: 10.1371/journal.pone.0337576 (PMC12680167; doi:10.1371/journal.pone.0337576)
Supplement: S2 Table — This table provides a comparative analysis between the newly proposed Node-Place-Jobs-housing (NPJ) classifications and the traditional Node-Place (NP) typologies for the 72 studied subway station areas. (DOC) [file pone.0337576.s002.doc]

| Table S2 | | | |
| --- | --- | --- | --- |
| Comparison of NPJ categories and NP types of 72 subway station areas in Density Zone 1 of Shenzhen | | | |
| Station area name | NPJ Category (Count) | NP Type (Count) | Proportion of NP Types within NPJ Category |
| Menghai Station | **Cluster 1**  **（6）** | Dependence  （4） | 67% |
| Pingshan Station |  |  |  |
| Linhai Station |  |  |  |
| Civic Center Station |  |  |  |
| Children's Palace Station |  | Unbalanced node  （2） | 33% |
| Guiwan Station |  |  |  |
| Xiangmihu Station | **Cluster 2**  **（35）** | Dependence  （2） | 6% |
| Xin'an Station |  |  |  |
| Chiwei Station |  | Balance  (24) | 69% |
| Universiade Center Station |  |  |  |
| Mangrove Bay Station |  |  |  |
| Huaqiang South Station |  |  |  |
| Jinlong Station |  |  |  |
| Liyumen Station |  |  |  |
| Lilin Station |  |  |  |
| Shangfen Station |  |  |  |
| Xinhe Station |  |  |  |
| Longcheng Park Station |  |  |  |
| Nanshan Book Mall Station |  |  |  |
| Bao an Station |  |  |  |
| Bao an Center Station |  |  |  |
| Baohua Station |  |  |  |
| Bao'an Stadium Station |  |  |  |
| Hongshan Station |  |  |  |
| Jixiang Station |  |  |  |
| Lianhuacun Station |  |  |  |
| Lianhua West Station |  |  |  |
| Ludancun Station |  |  |  |
| Nonglin Station |  |  |  |
| Shajing Station |  |  |  |
| Wenjin Station |  |  |  |
| Xiangmei Station |  |  |  |
| Dengliang Station |  | Unbalanced place  (9) | 26% |
| Longsheng Station |  |  |  |
| Shangtang Station |  |  |  |
| Shenwan station |  |  |  |
| Nanyou West Station |  |  |  |
| Yihai Station |  |  |  |
| Ailian Station |  |  |  |
| Huanggangcun Station |  |  |  |
| Liuhe Station |  |  |  |
| Hubei Station | **Cluster 3**  **（24）** | Stress  （13） | 54% |
| Nanshan Station |  |  |  |
| Huaxin Station |  |  |  |
| Science Museum Station |  |  |  |
| Grand Theater Station |  |  |  |
| Gangxia North Station |  |  |  |
| Pingshanwei Station |  |  |  |
| Shixia Station |  |  |  |
| Fumin Station |  |  |  |
| Renmin South station |  |  |  |
| Shaibu Station |  |  |  |
| Tongxinling Station |  |  |  |
| Xiangxicun station |  |  |  |
| Hongling South Station |  | Balance  (6) | 25% |
| Pingshan Square Station |  |  |  |
| Hongling Station |  |  |  |
| Houhai Station |  |  |  |
| Minzhi Station |  |  |  |
| Wonggekeng Station |  |  |  |
| Nanguang Station |  | Unbalanced place  (5) | 21% |
| Nanyou Station |  |  |  |
| Xiasha station |  |  |  |
| Guomao Station |  |  |  |
| Yannan station |  |  |  |
| Che Kung Temple Station | **Cluster 4**  **（7）** | Unbalanced node  （2） | 29% |
| Futian Railway Station |  |  |  |
| Gangxia Station |  | Stress  （5） | 71% |
| Shopping Park Station |  |  |  |
| Huaqiang Road Station |  |  |  |
| Laojie Station |  |  |  |
| Huaqiang North Station |  |  |  |
