# Supplementary figures and images for: Construction of the Node—place—Jobs-housing model: Analysis of employment-residential ratio in subway station areas of Shenzhen, China’s highest construction density zone
Source: PLoS One. 2025 Dec 5;20(12):e0337576. doi: 10.1371/journal.pone.0337576 (PMC12680167; doi:10.1371/journal.pone.0337576)

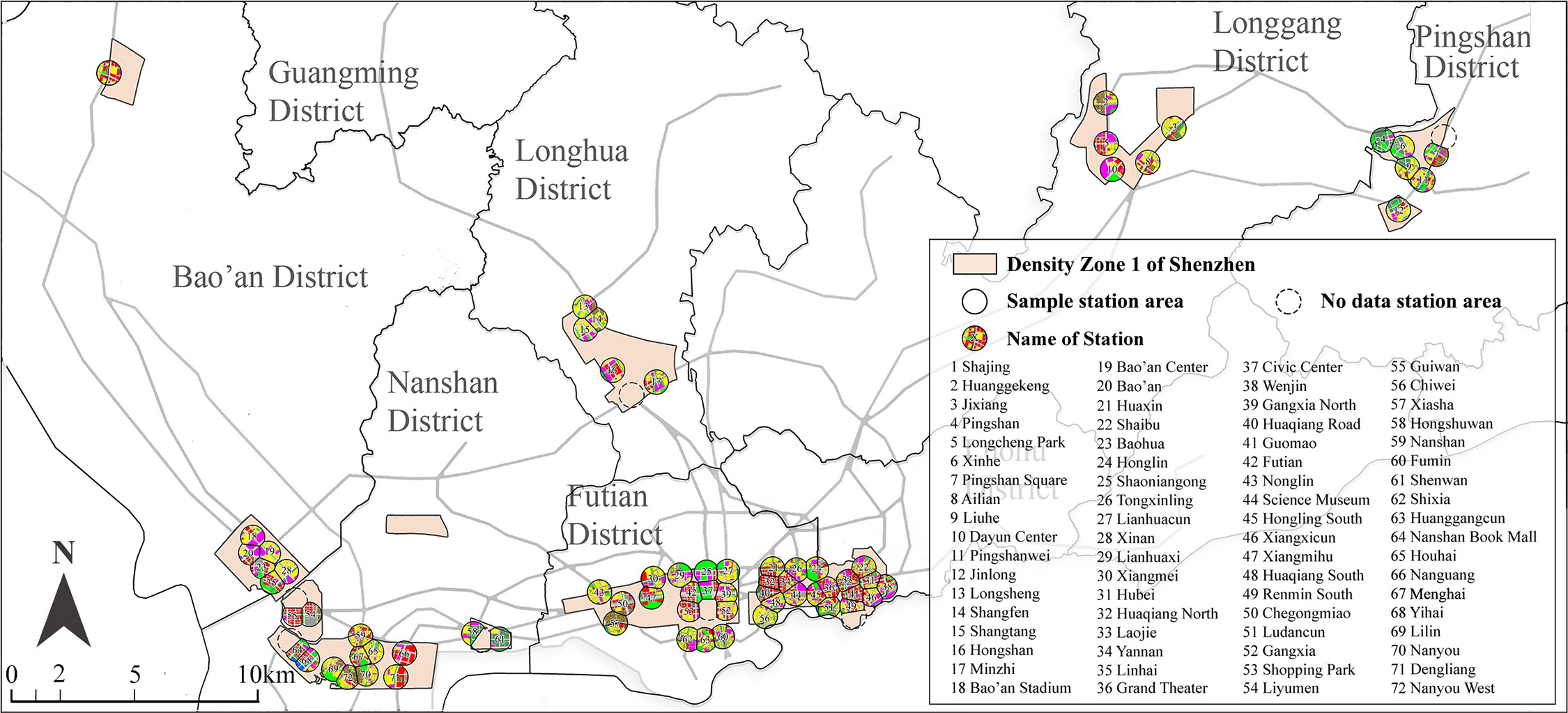

Supplement: S3 Fig — Base map data from OpenStreetMap. The figure is a simplified representation for illustrative purposes. This figure depicts the geographical locations of the 72 subway station areas under investigation, with each station being geographically labeled, within Shenzhen’s Density Zone 1. (TIF) [file pone.0337576.s003.tif]
