# Supplementary material for: Construction of the Node—place—Jobs-housing model: Analysis of employment-residential ratio in subway station areas of Shenzhen, China’s highest construction density zone
Source: PLoS One. 2025 Dec 5;20(12):e0337576. doi: 10.1371/journal.pone.0337576 (PMC12680167; doi:10.1371/journal.pone.0337576)
Supplement: S4 File — This file includes the complete results of normality tests (Kolmogorov-Smirnov and Shapiro-Wilk) and corresponding distribution histograms for Node values, Place values, Jobs-housing values, employment-residential area ratios, and employment-residential population ratios. (DOC) [file pone.0337576.s004.doc]

| Table S4(a) Normality test of Node values, Place values, and Jobs-housing values | | | | | | |
| --- | --- | --- | --- | --- | --- | --- |
|  | Kolmogorov-Smirnov^a^ | | | Shapiro-Wilk | | |
|  | Statistic | df | Sig. | Statistic | df | Sig. |
| Node | .078 | 72 | .200^*^ | .969 | 72 | .076 |
| Place | .062 | 72 | .200^*^ | .977 | 72 | .197 |
| Jobs-housing | .203 | 72 | .000 | .742 | 72 | .000 |
| *. This is a lower bound of the true significance. | | | | | | |
| a. Lilliefors Significance Correction | | | | | | |

| Table S4(b) Normality test of JH1(employment-residential area ratios) and JH1(employment-residential population ratios) | | | | | | |
| --- | --- | --- | --- | --- | --- | --- |
|  | Kolmogorov-Smirnova | | | Shapiro-Wilk | | |
|  | Statistic | df | Sig. | Statistic | df | Sig. |
| JH1 | .262 | 72 | .000 | .609 | 72 | .000 |
| JH2 | .207 | 72 | .000 | .730 | 72 | .000 |
| a. Lilliefors Significance Correction | | | | | | |

| 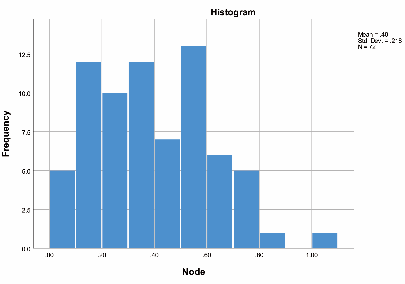 | 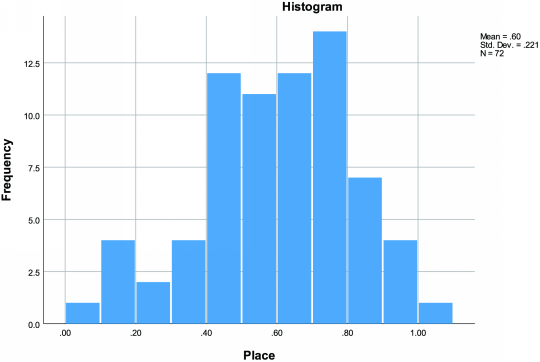 | | 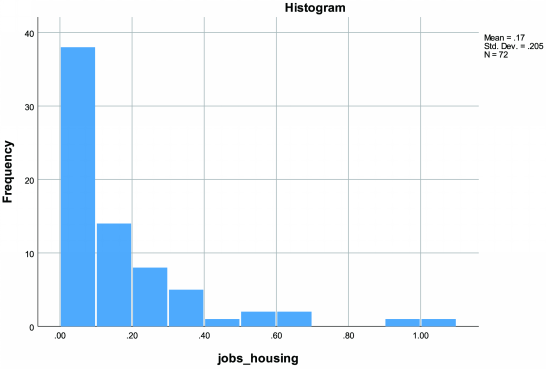 |
| --- | --- | --- | --- |
| Figure S4(a) Histogram of Node values | Figure S4(b) Histogram of Place values | | Figure S4(c) Histogram of Jobs-housing values |
| 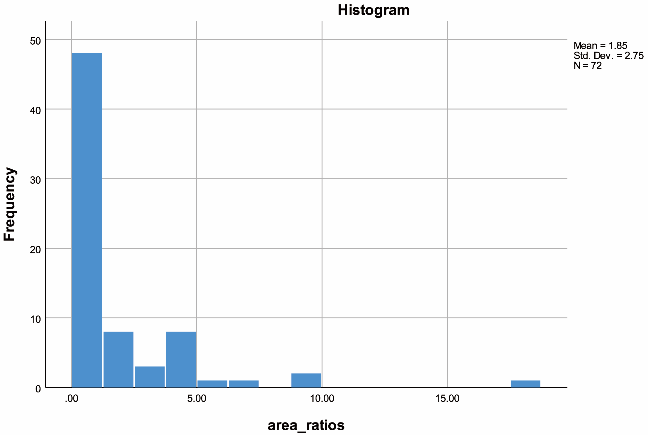 | | 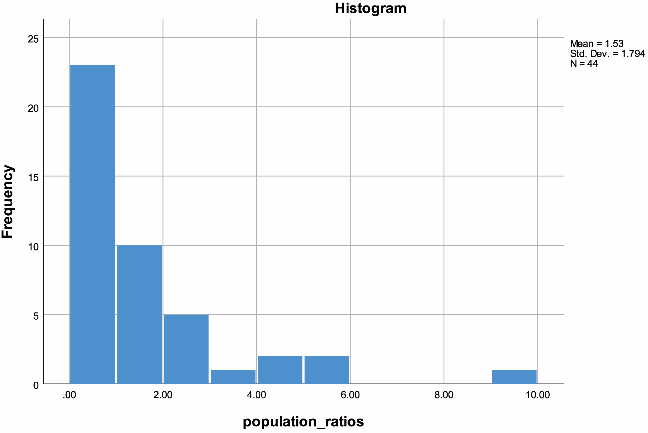 | |
| Figure S4(d) Histogram of employment-residential area ratios | | Figure S4(e) Histogram of employment-residential population ratios | |
| Figure S4 Histograms of Node values, Place values, Jobs-housing values, employment-residential area ratios, and employment-residential population ratios | | | |

Table S4(a) and Figure S4 present the normality test results and histograms for the Node, Place, and Jobs-housing values based on a sample size of 72 observations. The Kolmogorov-Smirnov test indicates that both Node values (D=0.078, p=0.200) and Place values (D=0.062, p=0.200) follow normal distributions, as evidenced by their bell-shaped histograms and statistically insignificant p-values (p>0.05). In contrast, the Jobs-housing values demonstrate a statistically significant deviation from normality (D=0.203, p=0.000), exhibiting a right-skewed distribution with a skewness coefficient of 2.185, where 72% of the data points cluster within the 0-0.2 range while containing a limited number of extreme high values.

Further normality tests were performed on the employment-residential area ratios and employment-residential population ratios within the Jobs-housing dimension. As presented in Table S4(a) and Figure S4, both ratios demonstrate positively skewed distributions. Specifically, the employment-residential area ratios show that 66.66% of samples fall within the 0-1.25 range and 26.38% within 1.25-5 range, with limited extreme high values observed. Similarly, the employment-residential population ratios indicate 54.17% of samples cluster in the 0-1 range and 36.11% in the 1.5-3 range, while containing few extreme values. These results consistently reveal non-normal distribution patterns for both ratio types under examination.
